# An Antibody‐CRISPR/Cas Conjugate Platform for Target‐Specific Delivery and Gene Editing in Cancer

**DOI:** 10.1002/advs.202308763

**Published:** 2024-03-29

**Authors:** Seungju Yang, San Hae Im, Ju Yeon Chung, Juhee Lee, Kyung‐Hun Lee, Yoo Kyung Kang, Hyun Jung Chung

**Affiliations:** ^1^ Department of Biological Sciences Korea Advanced Institute of Science and Technology Daejeon 34141 Republic of Korea; ^2^ Department of Internal Medicine Seoul National University Hospital Seoul 03080 Republic of Korea; ^3^ Cancer Research Institute Seoul National University Seoul 03080 Republic of Korea; ^4^ College of Pharmacy Gyeongsang National University Jinju 52828 Republic of Korea

**Keywords:** antibody conjugation, cancer therapy, CRISPR/Cas, in vivo gene editing, specific delivery

## Abstract

The CRISPR/Cas system has been introduced as an innovative tool for therapy, however achieving specific delivery to the target has been a major challenge. Here, an antibody‐CRISPR/Cas conjugate platform that enables specific delivery and target gene editing in HER2‐positive cancer is introduced. The CRISPR/Cas system by replacing specific residues of Cas9 with an unnatural amino acid is engineered, that can be complexed with a nanocarrier and bioorthogonally functionalized with a monoclonal antibody targeting HER2. The resultant antibody‐conjugated CRISPR/Cas nanocomplexes can be specifically delivered and induce gene editing in HER2‐positive cancer cells in vitro. It is demonstrated that the in vivo delivery of the antibody‐CRISPR/Cas nanocomplexes can effectively disrupt the *plk1* gene in HER2‐positive ovarian cancer, resulting in substantial suppression of tumor growth. The current study presents a useful therapeutic platform for antibody‐mediated delivery of CRISPR/Cas for the treatment of various cancers and genetic diseases.

## Graphical abstract

Here, an antibody‐CRISPR/Cas conjugate platform that enables specific delivery and target gene editing in HER2‐positive cancer is introduced. It is demonstrated that the in vivo delivery of the antibody‐CRISPR/Cas nanocomplexes can effectively disrupt the *plk1* gene in HER2‐positive ovarian cancer, resulting in substantial suppression of tumor growth.

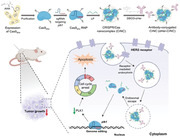

## 1 Introduction

Genome editing based on the clustered regularly interspaced short palindromic repeats/CRISPR‐associated protein (CRISPR/Cas) system has been shown promising for the treatment of human diseases, such as cancers and inherited genetic disorders.^[^ ^1^ ^]^ The disruption or correction of genes related to cell growth, survival, and resistance, can provide great advantages in cancer therapy by avoiding the need of frequent injections and preventing the chance of recurrence.^[^ ^2^ ^]^ However, application of the CRISPR/Cas system as an in vivo therapeutic for cancer has been challenging due to the poor delivery and selectivity to the target, leading to low efficacy and side effects.^[^ ^3^ ^]^ Various methods have been developed for the nonviral delivery of the CRISPR/Cas machinery, by using nanocarriers such as lipid formulations, polymers, and inorganic nanoparticles.^[^ ^4^ ^]^ For target‐specific delivery, rationally designed lipids or ligand conjugation on nanoparticles have been reported.^[^ ^5^ ^]^ However, the previous developments based on lipid chemistry have been mainly confined to RNA therapeutics, while antibody conjugation on surfaces can affect nano‐formulation stability and delivery efficiency.^[^ ^3^, ^6^ ^]^

For the delivery of the CRISPR/Cas ribonucleoproteins, chemical conjugation of biomolecules has been suggested as an alternative approach that can enable the desired delivery effects by fine‐tuning the molecular properties of the cargo.^[^ ^7^ ^]^ Particularly, the incorporation of unnatural amino acids into the CRISPR/Cas proteins can provide versatility in their modification by bioorthogonal chemistry, enabling multi‐functionalization and reducing by‐product formation.^[^ ^8^ ^]^ Previously, the CRISPR/Cas proteins Cas9 and Cas12 have been engineered by site‐specific incorporation of azido‐functionalized amino acids by strain‐promoted azide‐alkyne cycloadditions.^[^ ^7^, ^9^ ^]^ Further bioorthogonal modification of the unnatural amino acid‐incorporated Cas proteins with biomolecules such as carrier polymer, drug, or oligonucleotides has been shown to enable effective gene editing or combinatorial delivery.^[^ ^7^, ^9^ ^]^ A recent study has reported an engineered Cas9 that was site‐specifically incorporated with azidophenylalanine, followed by bioorthogonal conjugation with a chemotherapeutic drug and carrier polymer for self‐delivery and combinatorial therapy of cancer.^[^ ^7c^ ^]^ However, site‐specific incorporation techniques rely on the utilization of an amber codon, that can lead to premature termination of translation and the generation of truncated proteins.^[^ ^10^ ^]^ Furthermore, site‐specific incorporation of multiple amino acids to Cas9 can result in extremely low yield and purity of the protein, rendering it to be challenging to conjugate large molecules such as antibodies.^[^ ^11^ ^]^

**Figure 1:**
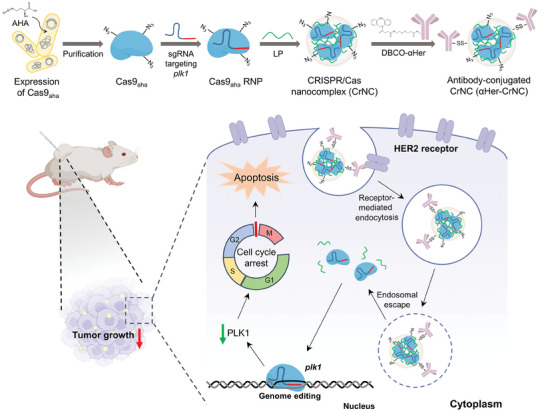
Schematic illustration on the development of the antibody‐CRISPR/Cas9 conjugate platform for target‐specific delivery and gene editing in cancer cells. The unnatural amino acid AHA is incorporated into Cas9 by replacing the Met residues to produce Cas9_aha_, and RNPs are formed by adding sgRNA targeting the *plk1* gene. The Cas9_aha_ RNPs are then complexed with a carrier polymer, LP, followed by reaction with DBCO‐functionalized anti‐HER2 antibody to form αHer‐CrNC. In vivo delivery of αHer‐CrNC to tumors results in *plk1* gene editing, leading to strong anti‐tumor effects.

Here, we developed an antibody‐conjugated CRISPR/Cas platform enabled by multivalent bioorthogonal functionalization, for target‐specific delivery and gene editing in vivo (**Figure** **1**). We engineered the Cas9 protein by residue‐specific incorporation of azidohomoalanine (AHA) that replaces the natural Met residues, and bioorthogonally conjugated with a monoclonal antibody specific to the HER2 receptor that is overexpressed in HER2‐positive cancer of the breast and ovaries.^[^ ^12^ ^]^ We demonstrate that with the assistance of a carrier polymer, nanocomplexes of the AHA‐incorporated Cas9 (Cas9_aha_) conjugated with the anti‐HER2 antibody (αHer‐CrNC = anti‐Her2 conjugated CRISPR nanocomplex) can be successfully delivered into HER2‐positive ovarian cancer cells with high selectivity, and disrupt the polo‐like kinase 1 (*plk1*) gene, leading to apoptosis of the target cells. Furthermore, the in vivo delivery of the αHer‐CrNC into mice tumors of HER2‐positive ovarian cancer can induce dramatic suppression of tumor growth (80.3%), showing potential as an effective gene editing therapeutic for cancer treatment.

## 2 Results and Discussion

### 2.1 Generation of Cas9 Incorporated with AHA

**Figure 2:**
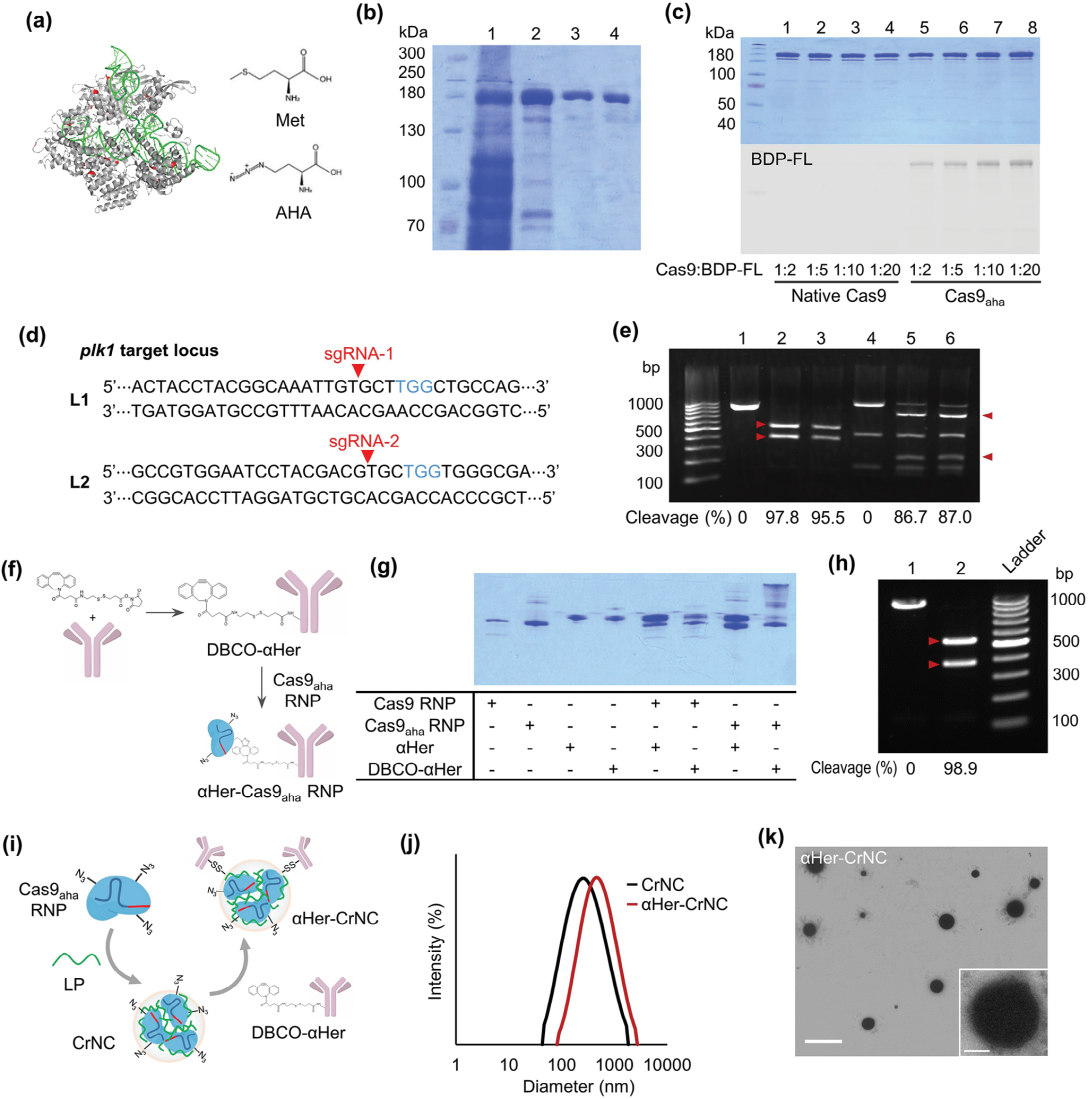
Preparation of Cas9_aha_ protein and antibody‐CRISPR/Cas conjugate complexes. a) Crystal structure of Cas9 with sgRNA (left, met residues colored in red), and chemical structures of Met and AHA (right). b) Purification of Cas9_aha_ (lane 1: B834(DE3) bacterial lysate; lane 2: pooled fraction after affinity chromatography; lane 3: pooled fraction after size exclusion chromatography; lane 4: purified Cas9_aha_ after dialysis). c) SDS‐PAGE analysis of purified Cas9_aha_ reacted with DBCO‐PEG_4_‐BDP‐FL, observed by coomassie blue staining (top) and fluorescence detection (bottom). d) Sequences of sgRNAs targeting the *plk1* gene, targeting two different loci, L1 (sgRNA‐1) and L2 (sgRNA‐2). e) DNA cleavage activities of Cas9_aha_ with sgRNAs targeting *plk1* (lanes 1,4: native Cas9 without sgRNA; lanes 2,5: native Cas9 RNP; lanes 3,6: Cas9_aha_ RNP). f) Schematic on the reaction of Cas9_aha_ RNPs with DBCO‐αHer, to form αHer‐Cas9_aha_ RNPs. g) SDS‐PAGE analysis of Cas9_aha_ RNPs reacted with DBCO‐αHer, that produces αHer‐Cas9_aha_ RNP. Native Cas9 (Cas9) and unmodified αHer were also used for the reactions as controls. h) DNA cleavage activities of αHer‐Cas9_aha_ RNPs including sgRNA‐1 (lane 1: αHer‐Cas9_aha_ without sgRNA‐1; lane 2: αHer‐Cas9_aha_ RNPs, including sgRNA‐1). i) Schematic on the preparation of αHer‐CrNC, that are αHer‐conjugated Cas9_aha_ RNPs complexed with LP, for carrier‐assisted delivery. j) DLS analysis of αHer‐CrNC, compared with unmodified Cas9_aha_ complexes. k) TEM analysis of αHer‐CrNC (scale bar: 2 µm, 200 nm).

We created a mutant version of Cas9 that is incorporated with the unnatural amino acid AHA (Cas9_aha_) as a bioorthogonal platform for versatile functionalization with biomolecules. Cas9 includes a total of 23 methionine (Met) residues, and among these 6 of them are exposed on the surface, according to structural prediction (**Figure** **2**a). We expected that the replacement of Met in Cas9 with AHA, an analogue for Met that includes azide functional groups, can endow bioorthogonal functionality to the protein for versatile modifications. We used the Met auxotroph B834(DE3), that relies on an external supply of Met for growth, to produce Cas9_aha_. The bacteria were not able to grow in Met‐depleted media, but grew slowly in the presence of AHA (Figure S1, Supporting Information). Purification of the protein gave rise to Cas9_aha_ with a purity of 95.9% (Figure 2b; Figure S2, Supporting Information). We examined the incorporation of AHA into Cas9 by first reacting the bacterial lysates with dibenzocyclooctyne‐functionalized BODIPY‐FL (DBCO‐BDP‐FL) to induce strain‐promoted azide‐alkyne cycloaddition, followed by gel electrophoresis. Fluorescence signals were detected in the bands including ones for Cas9 as well as the other expressed proteins, only for the lysates obtained from growth in presence of AHA, while signals were not detected for the lysates from growth in Met (Figure S3, Supporting Information). Reaction by copper‐mediated azide‐alkyne cycloaddition was also attempted by using alkyne‐BDP‐FL, revealing that bacterial lysates obtained by growth only in AHA‐ but not Met‐supplemented media resulted in the production of protein with reactivity by click chemistry (Figure S4, Supporting Information). Reaction of the purified Cas9_aha_ with DBCO‐BDP‐FL confirmed the bioorthogonal reactivity of the protein by AHA incorporation, as shown by the detection of fluorescence for Cas9_aha_ but not native Cas9 (Figure 2c). The increase in the molar ratio of Cas9_aha_ and DBCO‐BDP‐FL from 1:2 to 1:10 or higher resulted in saturation of fluorescence. Quantification of the AHA groups in Cas9 showed that an average of 5 molecules of AHA were incorporated into a single Cas9 molecule. We investigated the endonuclease activity of Cas9_aha_ using sgRNAs targeting the *plk1* gene and treating the RNPs to target DNA for *plk1* (Figure 2d). Results showed that Cas9_aha_ could cleave *plk1* target DNA with efficiencies of up to 95.5%, that was comparable to native Cas9 (97.8%) (Figure 2e). Moreover, Cas9_aha_ reacted with excess of DBCO‐AF647, and DBCO‐BDP‐FL did not cause any significant loss of endonuclease activity (Figure S5, Supporting Information). These results demonstrate that AHA was successfully incorporated into Cas9, and the resultant Cas9_aha_ as well as their functionalized forms via azide‐DBCO chemistry did not show any significant loss of endonuclease activity.

### 2.2 Preparation of Antibody‐Conjugated CRISPR/Cas Nanocomplexes

We attempted to establish a CRISPR/Cas nanocomplex formulation using bioorthogonal conjugation of the Cas9_aha_ ribonucleoprotein (RNP) with αHer, for target‐specific delivery. Since the conjugation of a large antibody molecule can disturb the endonuclease activity of Cas9, a disulfide bond was introduced so that the RNPs could be released from the antibodies into the cytosol after cell internalization. DBCO‐functionalized αHer (DBCO‐αHer) was generated by reacting the antibody with an NHS ester‐functionalized DBCO including a disulfide bond (Figure S6, Supporting Information). We then reacted Cas9_aha_ RNP with DBCO‐αHer to generate the αHer‐Cas9_aha_ RNPs (Figure 2f). A significant band shift was shown for Cas9_aha_ reacted with DBCO‐αHer, while the reaction with either the native Cas9 or the unmodified antibody did not (Figure 2g). Multiple bands were shown for Cas9_aha_ reacted with DBCO‐αHer, demonstrating the presence of Cas9‐antibody conjugates with a stoichiometry of single to multiple antibody molecules per Cas9 molecule. The conjugation efficiency of the αHer to Cas9_aha_ increased as the reaction time was increased (Figure S7a, Supporting Information). As the molar ratio of Cas9_aha_ RNP:DBCO‐αHer was increased from 1:1 to 1:2, the conjugation efficiency was increased from in 42.1% to 59.5%, while the efficiency decreased at even higher ratios (Figure S7b, Supporting Information). Increasing the reaction time also resulted in higher conjugation efficiency of up to 72.6%. At optimized conditions, the conjugation efficiency of αHer on Cas9_aha_ could reach up to 76.6%, that was 8.1–fold higher compared with the efficiency when native Cas9 was reacted with maleimide‐functionalized αHer (Mal‐αHer), that was 9.4% (Figure S7c, Supporting Information). Incubation of αHer‐Cas9_aha_ RNPs in reducing condition showed the antibody molecules could be successfully cleaved from the Cas9_aha_ RNPs (Figure S8, Supporting information). The cleavage activity of αHer‐Cas9_aha_ RNPs appeared to be 98.9% (Figure 2h), demonstrating that antibody conjugation did not affect the endonuclease activity of Cas9. This result demonstrates that the Cas9_aha_ platform enables robust and efficient conjugation with antibody molecules by bioorthogonal chemistry, without causing any significant loss of activity of Cas9.

We next prepared αHer‐conjugated CRISPR/Cas9 nanocomplexes (αHer‐CrNC) for delivery with the assistance of a carrier polymer (Figure 2i). We attempted the delivery of the Cas9_aha_ RNPs using various carrier materials, including polyethylenimines (linear and branched type), polyarginine, protamine, and the endosomolytic peptide ppTG21 (Figure S9, Supporting Information). We selected the cationic polymer linear polyethylenimine (Mw 10 kDa, = LP) as the carrier, as we anticipated that it can enable the most effective delivery, while causing less cytotoxicity compared to the other carriers, and the 20 kDa linear or 25 kDa branched PEI.^[^ ^13^ ^]^ The hydrodynamic diameter of the complexes was measured, showing values of 553.3 nm for the αHer‐CrNC, that was larger compared to the value of 350.0 nm shown for the unmodified Cas9_aha_ RNPs complexed with LP (CrNC) (Figure 2j). The polydispersity values of the αHer‐CrNC and control CrNC were 0.313 and 0.366, respectively. On the other hand, the hydrodynamic diameters of the controls without complexation with LP, αHer‐Cas9_aha_ RNPs and unmodified Cas9_aha_ RNPs, were 43.2 and 10.2 nm, respectively (Figure S10, Supporting Information). TEM analysis revealed the presence of particle‐like structures with sizes of ≈525.3 nm for αHer‐CrNC, while smaller particles resembling individual RNPs (<10 nm) were observed for CrNC (Figure 2k; Figure S11, Supporting Information). These results demonstrate the successful preparation of antibody‐CRISPR/Cas conjugates by bioorthogonal reaction of Cas9_aha_ with αHer, as well as the formation of nano‐sized complexes of the RNPs with a carrier polymer for potential delivery to cells.

### 2.3 Target Cell‐Specific Internalization of Antibody‐Conjugated CRISPR/Cas Nanocomplexes In Vitro

**Figure 3:**
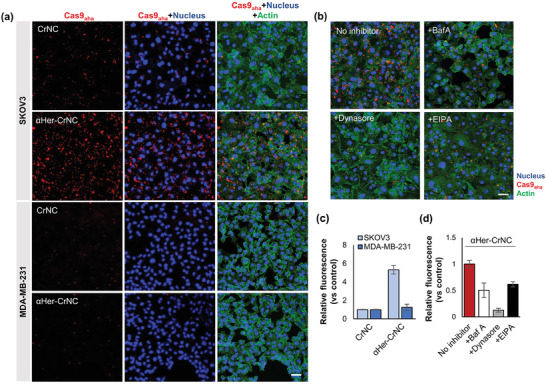
Target cell‐specific internalization of CRISPR/Cas nanocomplex by antibody‐mediated delivery. a) Confocal microscopy of HER‐positive (SKOV3) and HER2‐negative (MDA‐MB‐231) cancer cells treated with αHer‐CrNC (20X magnification, scale bar: 50 µm). b) Confocal images of SKOV3 cells treated with αHer‐CrNC in the presence of endocytosis inhibitors BafA, Dynasore, and EIPA. (20X magnification, scale bar: 50 µm) c) Quantification of fluorescence signals from images in a). d) Quantification of fluorescence from images in b) (Red: Cas9_aha_ labeled with AF647; blue: DAPI; green: actin).

We examined the selectivity of αHer‐CrNC delivered to HER2‐positive cancer cells in vitro. We first examined the expression level of HER2 in various cancer cell lines and validated that SKOV3 could express HER2 while MDA‐MB‐231 could not (Figure S12, Supporting Information). Then, we treated αHer‐CrNC that included Cas9_aha_ labeled with AF647 to SKOV3 ovarian cancer cells, that overexpresses HER2,^[^ ^14^ ^]^ and observed the level of fluorescence by confocal microscopy. **Figure** **3**a reveals that αHer‐CrNC were efficiently internalized into SKOV3 cells, shown by the strong fluorescence signal, compared to cells treated with control CrNC. However, when αHer‐CrNC were treated to cells that do not express HER2, MDA‐MB‐231, fluorescence could not be detected even after prolonged incubation (Figure S13, Supporting Information), indicating that the complexes could not be internalized. The treatment of αHer‐Cas9_aha_ RNPs without LP complexation to SKOV3 cells also resulted in significantly enhanced uptake compared to unmodified Cas9_aha_, whereas treatment to MDA‐MB‐231 cells did not (Figure S14, Supporting Information). The specific delivery to HER2‐positive cells was further demonstrated by preparing complexes conjugated with a non‐target antibody (αVim‐CrNC). Figure S15 (Supporting Information) reveals that αVim‐CrNC could not be efficiently internalized into SKOV3 cells, with signals being 33‐folds higher for αHer‐CrNC. These results demonstrate that the conjugation of αHer with the CRISPR/Cas nanocomplexes could drive the internalization of the complexes into HER2‐positive cancer cells with high selectivity.

We next investigated whether the delivery of αHer‐CrNC was mediated by receptor‐mediated endocytosis. We attempted the treatment of endocytosis inhibitors BafA, Dynasore, and EIPA, prior to treatment of αHer‐CrNC to SKOV3 cells. BafA and Dynasore are known as inhibitors of endosomal acidification and dynamin, respectively, while EIPA has been known as an inhibitor of plasma membrane Na+/H+ exchangers. The treatment of all three endocytosis inhibitors resulted in significant reductions of uptake of the αHer‐CrNC, as observed by confocal microscopy (Figure 3b). Among the inhibitors, Dynasore resulted in the strongest inhibition in cellular uptake, presumably by blocking vesicle scission during clathrin‐mediated endocytosis.^[^ ^15^ ^]^ Quantification of the signals showed that the relative fluorescence was increased to 5.3–folds for αHer‐CrNC compared to CrNC, in SKOV3 cells (Figure 3c), and lead to uptake efficiencies of 50.2%, 12.7%, and 61.6% for BafA, Dynasore, and EIPA, respectively, compared to without inhibitor treatment (Figure 3d). We treated αHer‐CrNC prepared with Cas9_aha_ and Herceptin labeled with fluorescein and AF647, respectively, to SKOVs cells and observed by confocal microscopy. Figure S16 (Supporting Information) shows that the fluorescence of Cas9_aha_ and Herceptin were located at different regions for αHer‐CrNC. On the other hand, when the non‐cleavable form of αHer‐CrNC (‐disulfide bond) was treated, co‐localization of Cas9_aha_ and Herceptin was observed in the cells. Treatment with CrNC+DBCO‐αHer resulted in very weak fluorescence of intracellular Cas9_aha_. This result supports that the antibody molecules could be released from the αHer‐CrNC, which can be important for the Cas9_aha_ RNPs to function in the cells. These results confirm that the internalization of αHer‐CrNC to HER2‐positive cancer cells occurred by receptor‐mediated endocytosis.

### 2.4 Gene Editing and Functional Analyses of Cancer Cells with αHer‐CrNC In Vitro

**Figure 4:**
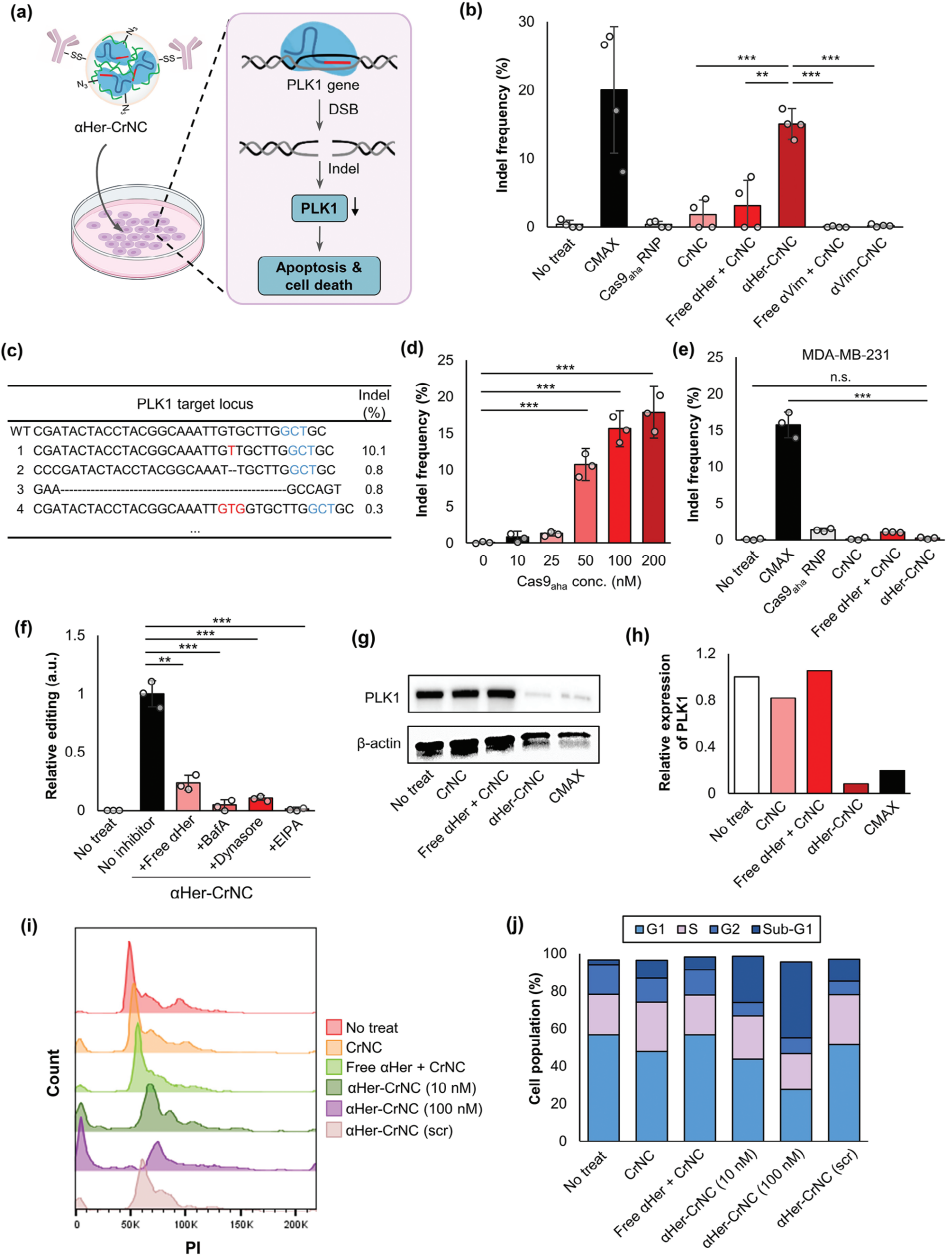
In vitro gene editing and functional analyses with αHer‐CrNC treatment. a) Schematic showing the effect of αHer‐CrNC on *plk1* disruption, leading to cellular apoptosis. b) Indel frequency of SKOV3 cells treated with αHer‐CrNC, compared with the lipofectamine formulation (CMAX), a mixture of CrNC and αHer (Free αHer + CrNC), CrNC conjugated with non‐target antibody (αVim‐CrNC), and other controls (*n* = 4). c) Representative sequences of *plk1* gene in SKOV3 cells treated with αHer‐CrNC (red: insertion, ‐: deletion, blue: PAM). Indel frequencies of d) SKOV3 cells treated with different concentrations of αHer‐CrNC (*n* = 3); e) MDA‐MB‐231 cells with the indicated treatments (*n* = 3); and f) SKOV3 cells treated with αHer‐CrNC in presence of free αHer or endocytosis inhibitors (*n* = 3). g) Western blot analysis of PLK1 protein in SKOV3 cells treated with αHer‐CrNC for 72 h, and h) relative quantitative analysis of expression levels. i) Cell cycle analysis of SKOV3 cells treated with αHer‐CrNC, and j) quantification of cell population (%) in each phase. Bars represent mean ± S.D. b,d,e,f) One‐way ANOVA, *:*p* <0.05, **:*p* <0.01, ***:*p* <0.001.

We assessed if the delivery of αHer‐CrNC can induce gene editing in target cells in vitro. αHer‐CrNC including sgRNA targeting the* plk1* gene were prepared, that can induce apoptosis and cell death upon disruption of *plk1* in HER2‐positive cancer cells (**Figure** **4**a). Targeted deep sequencing analysis of SKOV3 cells after treatment revealed that αHer‐CrNC could efficiently disrupt *plk1*, resulting in an average indel frequency of 15.1%, while CrNC with (Free αHer + CrNC) or without (CrNC) addition of free αHer resulted in indel frequencies of 4.1% and 2.4%, respectively (Figure 4b). Although the control CrNC could non‐specifically internalize to some extent into cells and lead to gene disruption, the results demonstrate that the effect was enhanced 8.5–folds upon conjugation with αHer. We further validated the selectivity of αHer‐CrNC on gene editing of SKOV3 cells by comparing with Cas9_aha_ RNPs complexed with LP and conjugated with a control antibody against vimentin (αVim‐CrNC). The indel frequency of cells treated with αVim‐CrNC targeting the *plk1* gene appeared to be below 0.2%, demonstrating that gene editing via internalization of αHer‐CrNC occurred by antibody‐antigen binding specific to HER2. Representative sequence data for αHer‐CrNC treated cells are shown in Figure 4c. Treatment of αHer‐CrNC to SKOV3 cells at various concentrations gave rise to an increase in indel frequency as the concentration of the complexes was increased (Figure 4d). We next examined the selectivity of *plk1* gene editing by treating αHer‐CrNC to MDA‐MB‐231 cells, that are negative for HER2. Figure 4e shows that a low indel frequency (0.2%) was observed in MDA‐MB‐231 cells treated with αHer‐CrNC, confirming that HER2‐specific delivery was achieved in the case of SKOV3 cells. When treated with control CrNC, non‐specific internalization was notably reduced in MDA‐MB‐231 cells compared to SKOV3 cells, that can be attributed to the different extent of internalization into the cell and nucleus, as well as chromatin remodeling at the target gene locus, depending on the cell type.^[^ ^17^ ^]^ Comparison of gene editing efficiencies by treatment of αHer‐conjugated Cas9_aha_ RNP complexes using various carriers including LP, ppTG21, polyarginine, and protamine showed that using LP resulted in significant gene editing, while the other carriers could not induce editing in SKOV3 cells (Figure S17, Supporting Information). We also assessed gene editing efficiency by the treatment of free αHer, and the endocytosis inhibitors BafA, Dynasore, and EIPA, prior to treating αHer‐CrNC. Figure 4f shows that the treatment of free αHer significantly reduced the editing efficiency of *plk1* by 75.1% compared to without free αHer. The pre‐treatment of the endocytosis inhibitors BafA, Dynasore, and EIPA resulted in a dramatic reduction in editing efficiency by 95.0%, 89.2%, and 98.6%, respectively, compared to without inhibitor treatment (No inhibitor, 13.9%). These results confirm that αHer‐CrNC can induce gene editing in the target cells upon delivery by selective binding to HER2 and subsequent receptor‐mediated endocytosis.

We next performed functional analysis on the effect of *plk1* gene editing by αHer‐CrNC on the regulation of the cell cycle. It has been known that *plk1* is a highly conserved serine‐threonine kinase that regulates G2/M transition, and has been a widely studied target for cancer therapy.^[^ ^16^ ^]^ Western blot analysis demonstrated that the treatment of αHer‐CrNC induced substantial downregulation of PLK1 expression in SKOV3 cells, compared to the unmodified CrNC and its mixture with free αHer (Figure 4g). Quantification of PLK1 expression level was reduced to ≈8% for αHer‐CrNC compared to control CrNC (Figure 4h). Treating the CRISPR/Cas nanocomplexes to NIH‐3T3 cells did not cause significant cytotoxicity nor apoptosis, even after treatment for 48 h (Figure S18, Supporting Information). Flow cytometry analysis revealed that *plk1* gene disruption by the delivery of αHer‐CrNC led to a significant increase in the population of the sub‐G1 phase (24.5% at 10 nM; 40.3% at 100 nM) (Figure 4i,j). On the other hand, control CrNC added with free αHer, and αHer‐CrNC including scrambled sgRNA (αHer‐CrNC (scr)) resulted in significantly lower sub‐G1 populations, that were 6.69%, and 11.4%, respectively. Taken together, these results demonstrate that the αHer‐CrNC can effectively induce apoptosis of HER2‐positive cancer cells by the disruption of *plk1*.

### 2.5 In Vivo Efficacy of αHer‐CrNC in a HER2‐Positive Ovarian Cancer Model

**Figure 5:**
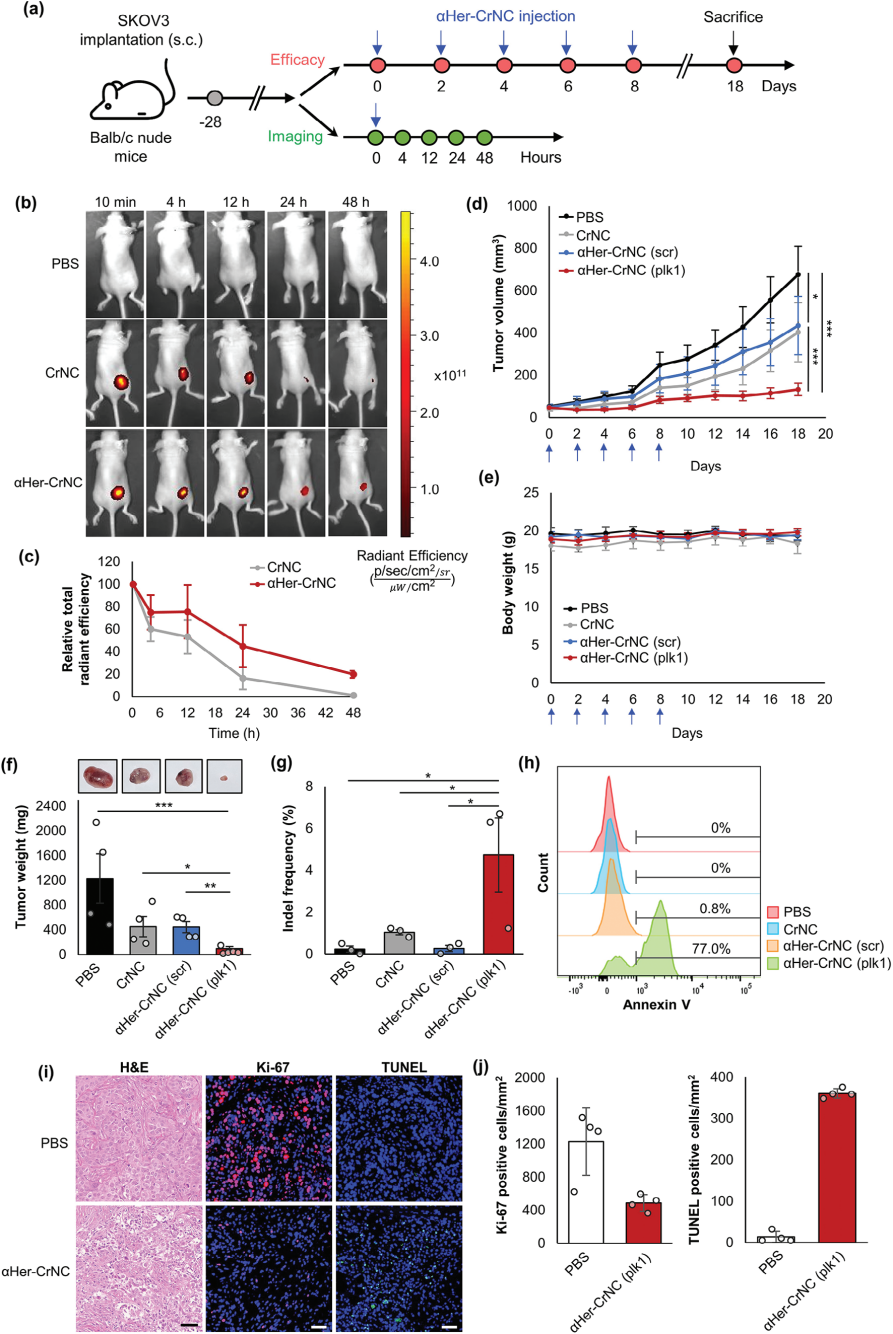
In vivo delivery and efficacy of αHer‐CrNC in an ovarian cancer model. a) Schematic of the timeline for in vivo delivery of αHer‐CrNC. SKOV3 cells were implanted (s.c.) into BALB/c nude mice, and when tumors were formed, αHer‐CrNC or the controls were injected intratumorally. b) Whole body imaging of αHer‐CrNC after single injection, by detecting the fluorescence of AF647 conjugated onto Cas9, and c) quantification of relative total radiant efficiencies. d–h) Anti‐tumor effect by αHer‐CrNC targeting *plk1*, after repeatedly injecting into tumors. d) Tumor volumes, and e) mouse body weights monitored every two days. f) Tumor weights and the representative tumor image for each group, after mice were sacrificed on day 18 (*n* = 4–5, One‐way ANOVA, *:*p* <0.05, **:*p* <0.01, ***:*p* <0.001). g) Indel frequency of tumors harvested from mice sacrificed on day 5 (*n* = 3, One‐way ANOVA, *:*p* <0.05, **:*p* <0.01, ***:*p* <0.001). h) Apoptosis levels of tumors measured by the Annexin V/PI assay. d–g) Bars represent mean ± S.E.M. i) Microscopic images of histological analyses of tumor tissues after treatment with the complexes by H&E, Ki‐67, and TUNEL staining (20X magnification, scale bar: 50 µm), and j) quantification of Ki‐67‐positive (left) and TUNEL‐positive (right) cells (bars represent mean ± S.D).

We evaluated the anti‐tumor efficacy of αHer‐CrNC upon in vivo delivery in a HER2‐positive ovarian cancer model. SKOV3 xenograft tumors were treated with αHer‐CrNC targeting *plk1* (αHer‐CrNC (plk1)) by repeated local injections, and the tumor growth was monitored (**Figure** **5**a). Imaging the in vivo fluorescence of the complexes labeled with AF647 revealed that αHer‐CrNC persisted in the tumor for a longer time compared to the control CrNC (Figure 5b). Quantification of fluorescence showed that the values were increased to 2.7‐folds at 24 h post‐treatment for αHer‐CrNC compared to the control CrNC (Figure 5c). To examine if αHer‐CrNC could target the tumor upon systemic delivery, we administered αHer‐CrNC once by intravenous injection to SKOV3 tumor‐bearing mice and observed the biodistribution. Figure S19 (Supporting Information) reveals significant accumulation of αHer‐CrNC in the tumor, while not for the controls, at 24 h post‐treatment. To evaluate therapeutic efficacy, αHer‐CrNC was administered by intratumoral injection and inhibition in tumor growth was assessed. Measuring the tumor volumes revealed that treating the αHer‐CrNC (plk1) complexes could significantly suppress tumor progression, showing 80.3% suppression on day 18 compared to the PBS control, whereas the unmodified CrNC and αHer‐CrNC (scr) controls lead to mild suppression that were 40.4% and 35.7%, respectively (Figure 5d). The average tumor volumes upon treatment of αHer‐CrNC (plk1) resulted in values of 133.2 mm^3^, while the control CrNC and αHer‐CrNC (scr) resulted in tumor volumes of 402.9 and 434.5 mm^3^, respectively. Average body weights of all treated mice did not significantly change during the monitored period (<1%), demonstrating that the treatment of αHer‐CrNC and the control CrNC did not cause any obvious side effects (Figure 5e). The average tumor weights on day 18 were 95.9 mg for the αHer‐CrNC (plk1) treatment group, whereas values of 449.9 and 444.2 mg were observed for the control CrNC and αHer‐CrNC (scr) controls (Figure 5f). These results show evidence on the in vivo efficacy of αHer‐CrNC (plk1) treatment, leading to strong anti‐tumor effects upon the specific delivery via binding to HER2 and disruption of *plk1*.

We next characterized the effect of αHer‐CrNC on gene editing by harvesting the tumor tissue on day 5 and analysis by targeted deep sequencing. Results showed that αHer‐CrNC (plk1) induced indels in the *plk1* gene of HER2‐positive tumor cells at a frequency of 4.7%, while the control CrNC and αHer‐CrNC (scr) groups did not induce significant gene editing, that were 1.0% and 0.2%, respectively (Figure 5g). The apoptotic effect of tumors by treatment of the complexes and gene editing was also characterized. Annexin V/PI staining and flow cytometry showed that αHer‐CrNC (plk1) could lead to a high level of apoptosis (77.0%), while the control CrNC and αHer‐CrNC (scr) groups did not, showing levels of 0% and 0.8%, respectively (Figure 5h; Figure S20, Supporting Information). To investigate the therapeutic mechanism of αHer‐CrNC, tumor tissues were stained by hematoxylin and eosin, Ki‐67, and terminal deoxynucleotidyl transferase dUTP nick end labeling (TUNEL) and observed (Figure 5i and j). The staining results revealed high levels of apoptosis/necrosis and inhibition of cell proliferation for the αHer‐CrNC treatment group, strongly supporting the results on tumor growth. In sum, these results demonstrate the strong anti‐tumor effects by the delivery of αHer‐CrNC, via significant gene disruption of *plk1*, leading to strong apoptotic effects of the tumor cells in vivo.

## 3 Conclusion

In summary, we developed the antibody‐CRISPR/Cas conjugate platform for target‐specific delivery based on a recombinant Cas9 that was incorporated with unnatural amino acids, Cas9_aha_. Nanocomplex formation and bioorthogonal reaction with an anti‐HER2 antibody was able to produce multivalent antibody‐CRISPR/Cas complexes (αHer‐CrNC), that was able to internalize into HER2‐positive cells by specific antibody‐antigen interaction. Target cell‐specific delivery of αHer‐CrNC complexed with a carrier polymer could induce significant gene editing of *plk1*, leading to apoptosis of the cells in vitro. Furthermore, in vivo delivery of αHer‐CrNC in a HER2‐positive ovarian cancer model could substantially suppress tumor growth, mediated by disruption of the *plk1* gene and cellular apoptosis in the tumors. Furthermore, αHer‐CrNC can be potentially applied even to undruggable targets and lead to sustained anti‐cancer effects, by the ability to precisely and permanently edit genes of target cells. The current study is the first report on an antibody‐CRISPR/Cas conjugate platform, providing a breakthrough for current gene editing therapeutics, that can resolve challenges in nonspecific delivery and off‐target effects in vivo.

## 4 Experimental Section

### Materials

Amino acids (Arg, His,Ile, Leu, Glu, Gly, Lys, Phe, Cys, Asp, Trp, Pro, Val, Ser, Thr, Tyr), M9 broth, and glucose were purchased from MBcell. Magnesium sulfate (MgSO_4_), calcium chloride (CaCl_2_), manganese chloride (MnCl_2_), iron chloride (FeCl_2_), ethyl‐isopropyl amiloride (EIPA), Dynasore, Bafilomycin A1 (BafA), linear polyethylenimine (Mw 10 kDa and 20 kDa), and branched polyethylenimine (Mw 25 kDa) were purchased from Sigma–Aldrich. AHA was purchased from BACHEM. DNeasy Blood and Tissue kit was purchased from Qiagen. Phusion High‐Fidelity DNA polymerase, Lipofectamine CRISPRMAX Cas9 Transfection Reagent, and sulfo‐succinimidyl‐4‐(N‐maleimidomethyl) cyclohexane‐1‐carboxylate (Sulfo‐SMCC) were purchased from Thermo Fisher. Expin PCR SV purification kit was obtained from GeneAll. All oligonucleotides were purchased from Macrogen (Table S1, Supporting Information). Herceptin was kindly provided from Dr. Kyung‐Hun Lee (Seoul National University Hospital). Azide‐Cyanine‐3 (Azide‐Cy3), dibenzocyclooctyne‐S‐S‐N‐hydroxysuccinimide ester (DBCO‐SS‐NHS ester), dibenzocyclooctyne‐Alexa Fluor 647 (DBCO‐Alexa Fluor 647), Alexa Fluor 647‐N‐hydroxysuccinimide ester (Alexa Fluor 647‐NHS ester), and Alexa Fluor 750‐N‐hydroxysuccinimide ester (Alexa Fluor 750‐NHS ester) were purchased from Click Chemistry Tool. Dibenzocyclooctyne‐tetraethylene glycol‐borondipyrromethene (DBCO‐PEG_4_‐BDP‐FL) and alkyne‐borondipyrromethene (alkyne‐BDP‐FL) were purchased from Jena Bioscience. Primary antibodies anti‐HER2 (D8F12, Cat#4290), anti‐PLK1 (208G4, Cat#4513, Lot#6) and anti‐Vimentin (D21H3, Cat#5741, Lot#6) were purchased from Cell Signaling. Anti‐β‐actin (C4, Cat#sc‐47778, Lot#H0522) was purchased from Santa Cruz Biotechnology. Horseradish peroxidase (HRP)‐conjugated goat‐anti‐mouse IgG antibody (Cat#1706516, Lot#64498211) and goat‐anti‐rabbit IgG (Cat#1706515, Lot#64354228) were purchased from Bio‐Rad. Propidium iodide and Annexin V solution for cell cycle analysis and apoptosis assay were obtained from BD Biosciences.

### Expression and Purification of Cas9_aha_

Competent cells of *E.coli* B834(DE3) (Merck, #69041), a methionine auxotroph, were transformed with pET28a vector including *Streptococcus pyogenes* Cas9 gene, 6x His, and nuclear localization sequence (Addgene, Plasmid #98158, see plasmid sequence in Table S2, Supporting Information). The bacteria were grown in Luri‐Bertani (LB) broth (BD Biosciences) including 100 µg mL^−1^ kanamycin at 37 °C overnight, and collected by centrifugation at 5000 g for 10 min at 4 °C. Pellets were re‐suspended in culture media (Table S3, Supporting Information) supplemented with 50 mg L^−1^ Met, and grown until OD_600_ = 0.8 at 37 °C. After centrifugation at 5000 g for 10 min at 4 °C, bacteria were re‐suspended in culture media, grown for 20 min at 37 °C, and added with 50 mg L^−1^ AHA and 0.5 mM isopropyl *β*‐d‐1‐thiogalactopyranoside (IPTG) for induction of Cas9_aha_. After growth for 16 h at 18 °C, bacteria were harvested by centrifugation at 5000 g for 10 min at 4 °C, treated with lysis buffer (300 mM NaCl, 50 mM NaH_2_PO_4_, 10 m imidazole, 0.1% Triton X‐100), and sonicated. The lysates were applied to a His‐tag column with Ni‐NTA agarose beads (GE healthcare). The eluent was then applied to size exclusion chromatography (Superdex 200 26/600, Cytiva) using AKTA Prime Plus. To characterize the reactivity of Cas9_aha_ in the bacterial lysates by click chemistry, the bacterial suspension after IPTG induction was added with an equal volume of PBS including Triton‐X‐100 (0.1%) and lysozyme (1 mg mL^−1^) for 30 min on ice, centrifuged, and the supernatant was reacted with 10 µM DBCO‐BDP‐FL at room temperature for 30 min. To characterize the reactivity of the purified Cas9_aha_, 1 µg of Cas9_aha_ was reacted with DBCO‐BDP‐FL (1:2 to 1:20 molar ratio) or alkyne‐BDP‐FL for 30 min at 37 °C. The reacted products were analyzed by sodium dodecyl sulfate‐polyacrylamide gel electrophoresis (SDS‐PAGE), and visualization using ChemiDoc (Bio‐Rad Laboratories).

### Preparation of αHer‐CrNC

For synthesis of DBCO‐αHer, 1 mg mL^−1^ of Herceptin in PBS (pH 8.0) was reacted with DBCO‐SS‐NHS ester at a molar ratio of 1:10 at room temperature for 2 h. The unreacted DBCO‐SS‐NHS ester were removed, and the buffer was changed to PBS (pH 7.4) using Amicon Ultra‐0.5 centrifugal filter (100 kDa) (Merck Millipore). For characterization, DBCO‐αHer was reacted with azide‐Cy3 at a molar ratio of 1:10 and 1:20, followed by SDS‐PAGE analysis and detection of fluorescence using ChemiDoc. For the preparing the αHer‐Cas9_aha_ RNPs, Cas9_aha_ RNPs were formed by mixing with sgRNA at 1:3 molar ratio and incubation at RT for 10 min, followed by reacting with DBCO‐αHer (molar ratio 1:1) at RT for 3 h, and dialysis using Slide‐A‐Lyzer Dialysis cassettes (MWCO 10 000; Thermo Fisher). For preparation of the αHer‐CrNC for delivery, Cas9_aha_ RNPs were complexed with 10 kDa LP at predetermined molar ratios, incubated at RT for 5 min, and purified with Amicon Ultra‐0.5 (100 kDa) to remove the uncomplexed LP. Then the Cas9_aha_ RNP complexed with LP was reacted with DBCO‐αHer (molar ratio 1:1) at room temperature for 3 h, to produce the αHer‐CrNC. The hydrodynamic sizes of the complexes were measured by ELSZ‐2000ZS (Otsuka). The conjugation efficiency of the final product was measured by SDS‐PAGE and image analysis using ImageJ.

### Cell Culture

SKOV3 ovarian cancer cells (Korean Cell Line Bank, KCLB#30 030) and MDA‐MB‐231 breast cancer cells were cultured in Roswell Park Memorial Institute (RPMI) 1640 (Hyclone) supplemented with 10% fetal bovine serum (Gibco), L‐glutamine (Thermo Fisher), 25 mM 4‐(2‐hydroxyethyl)−1‐piperazineethanesulfonic acid (HEPES), and 1% penicillin/streptomycin (P/S, Gibco) in an atmosphere of 5% CO_2_ at 37 °C (BB15, Thermo Fisher).

### Endonuclease Activity

Target DNA for the *plk1* gene was amplified from the genomic DNA extracted from cultured SKOV3 cells using specific primers (Table S1, Supporting Information). One hundred and twenty nanograms of *plk1* target DNA was added with Cas9_aha_ or native Cas9 RNPs including sgRNA for *plk1* and incubated at 37 °C for 90 min in 3.1 buffer (NEB). The mixture was inactivated by adding RNase A, 20% SDS, and 0.5 M EDTA. The cleavage product was analyzed by 1% agarose gel electrophoresis. Cleavage efficiency was calculated using the following formula:

(1) $$\begin{array}{ccc} \mathrm{Cleavage}\left(\%\right) & = & \left(\mathrm{Intensity}\;\;\mathrm{of}\;\;\mathrm{cleaved}\;\;\mathrm{band}\right)/\\ & & \left(\mathrm{Intensity}\;\;\mathrm{of}\;\;\mathrm{sum}\;\;\mathrm{of}\;\;\mathrm{cleaved}\;\;\mathrm{and}\;\;\mathrm{uncleaved}\;\;\mathrm{band}\right)\;\times 100 \end{array}$$

The intensities of each band were quantified using ImageJ.

### Cellular Uptake

A total of 4 × 10^4^ SKOV3 cells were seeded in 8‐well chamber slides (SPL Life Sciences), and cultured overnight for cell attachment. αHer‐Cas9_aha_ or αHer‐CrNC (30 nm) was then treated to the cells for 4 h, washed twice with PBS, and fixed with 4 v/v% formaldehyde solution. Cells were then permeabilized with 0.1 v/v% Triton X‐100, and washed twice with PBS. For actin staining, cells were added with ActinRed 555 (Invitrogen) and incubated at RT for 30 min. Cells were then mounted using VectaShield including DAPI (Vector Laboratories) and observed by confocal microscopy (LSM 880, Carl Zeiss). For endocytosis inhibitor treatment, 50 µM EIPA, 80 µM Dynasore, or 100 nM BafA were added for 30 min prior to treating αHer‐CrNC, and processed as described above. The efficiency of cellular uptake was determined using Zen Blue (Carl Zeiss).

### Western Blot

To examine HER2 or PLK1 expression by western blot, SKOV3 cells (1.6 × 10^5^) were seeded in a 6‐well plate, and cultured overnight. The cells were treated with the αHer‐CrNC or the controls for 72 h, harvested, and lysed in RIPA buffer including protease/phosphatase inhibitor (Cell Signaling). Total protein amount of the lysates was quantified by the bicinchoninic acid (BCA, Thermo Fisher) assay, and 40 µg of protein were separated by 7.5% SDS‐PAGE followed by transfer to a polyvinylidene difluoride (PVDF) membrane. After the membrane was incubated with anti‐HER2, anti‐PLK1 or anti‐β‐actin antibodies at 4 °C overnight, the membrane was rinsed, and incubated with goat anti‐rabbit IgG‐HRP or goat anti‐mouse IgG‐HRP at RT for 1 h. The bands were detected by adding ECL (Bio Rad) and visualization using ChemiDoc.

### Gene Editing of plk1 In Vitro

Cells (4 × 10^4^) were seeded in a 24‐well plate, cultured overnight, and treated with the αHer‐CrNC or the controls at an atmosphere of 5% CO_2_ at 37 °C for 48 h. Cells were harvested and genomic DNA were extracted using DNeasy Blood and Tissue kit. Target genes were amplified by PCR using specific primers for the target *plk1* gene (Table S1, Supporting Information) and next‐generation sequencing was performed using MiniSeq (Illumina). The indel frequencies were evaluated using an online‐tool Cas‐Analyzer (http://www.rgenome.net/cas‐analyzer/#!).

### Cytotoxicity Assay

Cell viability was examined using the Cell Counting Kit‐8 (CCK‐8 assay, Dojindo Molecular Technologies). SKOV3 cells (1 × 10^4^) were seeded in a 96‐well plate, cultured overnight, and treated with the complexes at an atmosphere of 5% CO_2_ at 37 °C for pre‐determined times. Cells were washed with culture media, treated with 10 µL of water‐soluble tetrazolium 8, and incubated at 37 °C for 90 min. The absorbance at 450 nm was measured with Infinite M200 PRO (Tecan), and cell viability was determined based on normalization with values for the control (no treatment).

### Cell Cycle Analysis

SKOV3 cells (8 × 10^4^) were seeded in a 24‐well culture plate, incubated for 24 h at 37 °C, and washed with fresh media. Cells were then treated with 100 nM of αHer‐CrNC for 72 h, washed with cold DPBS, and harvested. Cells were fixed by gently dropping cold EtOH (Sigma–Aldrich) into the cell suspension up to 70% v/v, incubated for 30 min on ice, and then washed with DPBS. After resuspending in DPBS, cells were added with 10 ug mL^−1^ RNase A, incubated for 30 min at RT, and stained with PI. Analysis was performed using FACS LSRFortessa (BD Biosciences), and processed using FlowJo.

### In Vivo Studies

Female 4‐week‐old BALB/c nude mice were purchased from OrientBio. All animal studies were performed following authorized protocols and in accordance with the policies of the Institutional Animal Care and Use Committee (IACUC) of KAIST, with protocol no. KA2021‐103. For generating SKOV3 xenograft tumors in mice, female 5‐week‐old BALB/c nude mice were anesthetized using isoflurane exposure, and 1.5 × 10^6^ SKOV3 cells suspended in 200 µL of PBS containing 50% Matrigel Basement Membrane Matrix, Phenol Red‐free (Corning) were implanted subcutaneously into the dorsal flanks of female 5‐week‐old BALB/c nude mice. When tumor volumes reached ≈45 mm^3^,^[^ ^18^ ^]^ mice were randomly divided into different treatment groups and the experiments were performed. For whole body imaging, mice (*n* = 2) were treated once with the complexes or controls, and the fluorescence of AF647 conjugated onto Cas9_aha_ was measured at various time points using IVIS Lumina S5 (Perkin Elmer). For biodistribution, mice (*n* = 2) were administered AF750 conjugated αHer‐CrNC once by intravenous injection and sacrificed at 24 h post‐treatment. Fluorescence of each organs and tumor were measured using IVIS Lumina S5. For measuring tumor growth, each group (*n* = 4‐5; PBS, CrNC, αHer‐CrNC (scr), and αHer‐CrNC (plk1)) was intratumorally treated with the complexes (20 µg of Cas9_aha_ per 30 µL PBS) or the control, 5 times every two days. Tumor volumes and mouse body weights were measured every two days. To assess gene editing of *plk1* in mice tumors, mice were treated (*n* = 3) with the complexes as for the tumor growth experiment, sacrificed by carbon dioxide inhalation at day 5, and tumor tissues were harvested. Genomic DNA was extracted from the tumors using QIAamp Fast DNA Tissue Kit (Qiagen) and target genes were amplified by PCR, and next‐generation sequencing was performed using MiniSeq. For measuring apoptosis, three mice in each group were sacrificed by carbon dioxide inhalation on day 18, and tumors were harvested. Tumor tissues were fixed by 10% formalin solution for 1 h, chopped into 1–3 mm^3^ pieces, followed by incubation with 1 mg mL^−1^ collagenase IV and 100 Kunitz mL^−1^ DNase I for 1 h in an atmosphere of 5% CO_2_ at 37 °C. Cells were isolated using a cell strainer (70 µm, Corning), added with ACK lysis buffer, and stained with Annexin V and PI for analysis using FACS LSRFortessa and FlowJo. For histological staining, αHer‐CrNC (plk1) was treated to SKOV3 xenograft tumors (n = 2, 20 µg of Cas9_aha_ per 30 µL PBS) 5 times every two days, and tumor tissues were harvested at day 9. Tissues were sectioned followed by H&E, Ki‐67, and TUNEL staining, and observed by microscopy.

### Statistical Analysis

All statistical analyses were conducted using GraphPad Prism. All assay experiments were performed in triplicates or quadruplicates. Data were presented as mean ± standard deviation or mean ± standard error. *p* values were obtained using One‐way ANOVA. A *p* value of <0.05 was considered statistically significant (*p ≤ 0.05, **p ≤ 0.01, ***p ≤ 0.001).

## Conflict of Interest

The authors declare no conflict of interest.

## Supporting information

Supporting Information

## Data Availability

The data that support the findings of this study are available from the corresponding author upon reasonable request.
