# Supplementary material for: An Antibody‐CRISPR/Cas Conjugate Platform for Target‐Specific Delivery and Gene Editing in Cancer
Source: Adv Sci (Weinh). 2024 Mar 29;11(21):2308763. doi: 10.1002/advs.202308763 (PMC11151032; doi:10.1002/advs.202308763)
Supplement: Supplementary file 1 — Supporting Information [file ADVS-11-2308763-s001.pdf]

ADVANCED SCIENCE
Open Access

Supporting Information

for *Adv. Sci.*, DOI 10.1002/advs.202308763

An Antibody-CRISPR/Cas Conjugate Platform for Target-Specific Delivery and Gene Editing in Cancer

*Seungju Yang, San Hae Im, Ju Yeon Chung, Juhee Lee, Kyung-Hun Lee, Yoo Kyung Kang and Hyun Jung Chung**

Supporting Information

An Antibody-CRISPR/Cas Conjugate Platform for Target-Specific Delivery and Gene Editing in Cancer

*Seungju Yang, San Hae Im, Ju Yeon Chung, Juhee Lee, Kyung-Hun Lee, Yoo Kyung Kang, Hyun Jung Chung**

Table S1. Sequences of oligonucleotides for target DNA, sgRNAs, and primers.

Type of oligonucleotide	Sequences of oligonucleotides
PLK1 gene-1_Fwd	GGAGTACCCAGGGAGGAGTC
PLK1 gene-1_Rev	GCTGCCCTAAGAAAATGCTG
PLK1 gene-2_Fwd	AAGAGATCCCGGAGGTCCTA
PLK1 gene-2_Rev	GGAAGGGAGAGGGTTACCAG
PLK1 gene_adaptor_Fwd	ACACTCTTTCCCTACACGACGCTCTTCCGATCTCTTCCCACCCACAGTCTCTC
PLK1 gene_adaptor_Rev	GTGACTGGAGTTCAGACGTGTGCTCTTCCGATCTTTCAGGAAAAGGTTGCCCAG
sgRNA-1	GAAATTAATACGACTCACTATAGGTACCTACGGCAAATTGTGCTGTTTTAGAGCTAGAAATAGCAAGTTAAAATAAGGCTAGTCCGTTATCAACTTGAAAAAGTGGCACCGAGTCGGTGCTTTTTT
sgRNA-2	GAAATTAATACGACTCACTATAGGCGTGGAATCCTACGACGTGCGTTTTAGAGCTAGAAATAGCAAGTTAAAATAAGGCTAGTCCGTTATCAACTTGAAAAAGTGGCACCGAGTCGGTGCTTTTTT

Table S2. Sequence of pET28a-Cas9-His plasmid.

Plasmid	Sequences
pET28a-Cas9-His (9387 bp)	CCCATTCGCCAATCCGGATATAGTTCCTCCTTTCAGCAAAAAACCCCTCAAGACCCGTTTAGAGGCCCC<br>AAGGGGTTATGCTAGTTATTGCTCAGCGGTGGCAGCAGCCAACTCAGCTTCCTTTCGGGCTTTGTTAG<br>CAGCCGGATCTCAGTGGTGGTGGTGGTGGTGCTCGAGCACCTTCCTCTTCTTCTTGGGGTCACCTCCT<br>AGCTGACTCAAATCAATGCGTGTTTCATAAAGACCAGTGATGGATTGATGGATAAGAGTGGCATCTAAA<br>ACTTCTTTTGTAGACGTATATCGTTTACGATCAATTGTTGTATCAAAATATTTAAAAGCAGCGGGAGCTC<br>CAAGATTCGTCAACGTAAATAAATGAATAATATTTTCTGCTTGTTCACGTATTGGTTTGTCTCTATGTTTG<br>TTATATGCACTAAGAACTTTATCTAAATTGGCATCTGCTAAAATAACACGCTTAGAAAATTCACTGATTTG<br>CTCAATAATCTCATCTAAATAATGCTTATGCTGCTCCACAAACAATTGTTTTTGTTCGTTATCTTCTGGAC<br>TACCCTTCAACTTTTCATAATGACTAGCTAAATATAAAAAATTCACATATTTGCTTGGCAGAGCCAGCTC<br>ATTTCCTTTTTGTAATTCTCCGGCACTAGCCAGCATCCGTTTACGACCGTTTTCTAACTCAAAAAGACTA<br>TATTTAGGTAGTTTAATGATTAAGTCTTTTTTAACTTCCTTATATCCTTTAGCTTCTAAAAAGTCAATCGG<br>ATTTTTTTCAAAGGAACTTCTTTCCATAATTGTGATCCCTAGTAACTCTTTAACGGATTTTAACTTCTTCG<br>ATTTCCCTTTTTCCACCTTAGCAACCACTAGGACTGAATAAGCTACCGTTGGACTATCAAAACCACCATA<br>TTTTTTTGGATCCCAGTCTTTTTTACGAGCAATAAGCTTGTCCGAATTTCTTTTTGGTAAAATTGACTCCT<br>TGGAGAATCCGCCTGTCTGTACTTCTGTTTTCTTGACAATATTGACTTGGGGCATGGACAATACTTTGC<br>GCACTGTGGCAAAATCTCGCCCTTTATCCCAGACAATTTCTCCAGTTTCCCCATTAGTTTCGATTAGAG<br>GGCGTTTGCGAATCTCTCCATTTGCAAGTGTAATTTCTGTTTTGAAGAAGTTCATGATATTAGAGTAAAA<br>GAAATATTTTGCGGTTGCTTTGCCTATTTCTTGCTCAGACTTAGCAATCATTTTACGAACATCATAAACTT<br>TATAATCACCATAGACAAACTCCGATTCAAGTTTTGGATATTTCTTAATCAAAGCAGTTCCAACGACGGC<br>ATTTAGATACGCATCATGGGCATGATGGTAATTGTTAATCTCACGTACTTTATAGAATTGGAAATCTTTT<br>CGGAAGTCAGAAACTAATTTAGATTTTAAGGTAATCACTTTAACCTCTCGAATAAGTTTATCATTTTCATC<br>GTATTTAGTATTCATGCGACTATCCAAAATTTGTGCCACATGCTTAGTGATTTGGCGAGTTTCAACCAAT<br>TGGCGTTTGATAAAACCAGCTTTATCAAGTTCACTCAAACCTCCACGTTCAGCTTTCGTTAAATTATCAA<br>ACTTACGTTGAGTGATTAACTTGGCGTTTAGAAGTTGTCTCCAATAGTTTTTCATCTTTTTGACTACTTCT<br>TCACTTGGAACGTTATCCGATTTACCACGATTTTTATCAGAACGCGTTAAGACCTTATTGTCTATTGAAT<br>CGTCTTTAAGGAAACTTTGTGGAACAATGTGATCGACATCATAATCACTTAAACGATTAATATCTAATTC<br>TTGGTCCACATACATGTCTCTTCCATTTTGGAGATAATAGAGATAGAGCTTTTCATTTTGCAATTGAGTA<br>TTTTCAACAGGATGCTCTTTAAGAATCTGACTTCCTAATTCTTTGATACCTTCTTCGATTCGTTTCATACG<br>CTCTCGCGAATTTTTCTGGCCCTTTTGAGTTGTCTGATTTTCACGTGCCATTTCAATAACGATATTTTCT<br>GGCTTATGCCGCCCCATTACTTTGACCAATTCATCAACAACTTTTACAGTCTGTAAAATACCTTTTTTAAT<br>AGCAGGGCTACCAGCTAAATTTGCAATATGTTCATGTAAACTATCGCCTTGTCCAGACACTTGTGCTTTT<br>TGAATGTCTTCTTTAAATGTCAAACTATCATCATGGATCAGCTGCATAAAATTGCGATTGGCAAAACCAT<br>CTGATTTCAAAAAATCTAATATTGTTTTGCCAGATTGCTTATCCCTAATACCATTAATCAATTTTCGAGAC<br>AAACGTCCCCAACCAGTATAACGGCGACGTTTAAGCTGTTTCATCACCTTATCATCAAAGAGGTGAGCA<br>TATGTTTTAAGTCTTTCCTCAATCATCTCCCTATCTTCAAATAAGGTCAATGTTAAAACAATATCCTCTAA<br>GATATCTTCATTTTCTTCATTATCCAAAAAATCTTTATCTTTAATAATTTTTAGCAAATCATGGTAGGTACC<br>TAATGAAGCATTAAATCTATCTTCAACTCCTGAAATTTCAACACTATCAAAACATTCTATTTTTTTGAAATA<br>ATCTTCTTTTAATTGCTTAACGGTTACTTTTCGATTTGTTTTGAAGAGTAAATCAACAATGGCTTTCTTCT<br>GTTCACCTGAAAGAAATGCTGGTTTTCGCATTCCTTCAGTAACATATTTGACCTTTGTCAATTCGTTATA<br>AACCGTAAAATACTCATAAAGCAAACTATGTTTTGGTAGTACTTTTTCATTTGGAAGATTTTTATCAAAGT<br>TTGTCATGCGTTCAATAAATGATTGAGCTGAAGCACCTTTATCGACAACTTCTTCAAAATTCCATGGGGT<br>AATTGTTTCTTCAGACTTCCGAGTCATCCATGCAAAACGACTATTGCCACGCGCCAATGGACCAACATA<br>ATAAGGAATTCGAAAAGTCAAGATTTTTTCAATCTTCTCACGATTGTCTTTTAAAAATGGATAAAAGTCTT<br>CTTGTCTTCTCAAAATAGCATGCAGCTCACCCAAGTGAATTTGATGGGGAATAGAGCCGTTGTCAAAGG<br>TCCGTTGCTTGCGCAGCAAATCTTCACGATTTAGTTTCACCAATAATTCCTCAGTACCATCCATTTTTTC<br>TAAAATTGGTTTGATAAATTTATAAAATTCTTCTTGGCTAGCTCCCCCATCAATATAACCTGCATATCCGT<br>TTTTTGATTGATCAAAAAAGATTTCTTTATACTTTTCTGGAAGTTGTTGTCGAACTAAAGCTTTTAAAAGA<br>GTCAAGTCTTGATGATGTTCATCGTAGCGTTTAATCATTGAAGCTGATAGGGGAGCCTTAGTTATTTCA<br>GTATTTACTCTTAGGATATCTGAAAGTAAAATAGCATCTGATAAATTCTTAGCTGCCAAAAACAAATCAG<br>CATATTGATCTCCAATTTGCGCCAATAAATTATCTAAATCATCATCGTAAGTATCTTTTGAAAGCTGTAAT<br>TTAGCATCTTCTGCCAAATCAAAATTTGATTTAAAATTAGGGGTCAAACCCAATGACAAAGCAATGAGAT<br>TCCCAAATAAGCCATTTTTCTTCTCACCGGGGAGCTGAGCAATGAGATTTTCTAATCGTCTTGATTTACT<br>CAATCGTGCAGAAAGAATCGCTTTAGCATCTACTCCACTTGCGTTAATAGGGTTTTCTTCAAATAATTGA<br>TTGTAGGTTTGTACCAACTGGATAAATAGTTTGTCCACATCACTATTATCAGGATTTAAATCTCCCTCAA<br>TCAAAAAATGACCACGAAACTTAATCATATGCGCTAAGGCCAAATAGATTAAGCGCAAATCCGCTTTAT<br>CAGTAGAATCTACCAATTTTTTTCGCAGATGATAGATAGTTGGATATTTCTCATGATAAGCAACTTCATC<br>TACTATATTTCCAAAAATAGGATGACGTTCATGCTTCTTGTCTTCTTCCACCAAAAAAGACTCTTCAAGT<br>CGATGAAAGAAACTATCATCTACTTTCGCCATCTCATTTGAAAAAATCTCCTGTAGATAACAAATACGAT<br>TCTTCCGACGTGTATACCTTCTACGAGCTGTCCGTTTGAGACGAGTCGCTTCCGCTGTCTCTCCACTGT<br>CAAATAAAAGAGCCCCTATAAGATTTTTTTTGATACTGTGGCGGTCTGTATTTCCCAGAACCTTGAACTT<br>TTTAGACGGAACCTTATATTCATCAGTGATCACCGCCCATCCGACGCTATTTGTGCCGATATCTAAGCC<br>TATTGAGTATTTCTTATCCATCACCTTCCTCTTCTTCTTGGGCATAAACCCCATGGTATATCTCCTTCTTA<br>AAGTTAAACAAAATTATTTCTAGAGGGGAATTGTTATCCGCTCACAATTCCCCTATAGTGAGTCGTATTA<br>ATTTCGCGGGATCGAGATCTCGATCCTCTACGCCGGACGCATCGTGGCCGGCATCACCGGCGCCACA<br>GGTGCGGTTGCTGGCGCCTATATCGCCGACATCACCGATGGGGAAGATCGGGCTCGCCACTTCGGGC<br>TCATGAGCGCTTGTTTCGGCGTGGGTATGGTGGCAGGCCCCGTGGCCGGGGGACTGTTGGGCGCCA<br>TCTCCTTGCATGCACCATTCCTTGCGGCGGCGGTGCTCAACGGCCTCAACCTACTACTGGGCTGCTTC<br>CTAATGCAGGAGTCGCATAAGGGAGAGCGTCGAGATCCCGGACACCATCGAATGGCGCAAAACCTTT<br>CGCGGTATGGCATGATAGCGCCCGGAAGAGAGTCAATTCAGGGTGGTGAATGTGAAACCAGTAACGT<br>TATACGATGTCGCAGAGTATGCCGGTGTCTCTTATCAGACCGTTTCCCGCGTGGTGAACCAGGCCAG

Table S2 (*continued*). Sequence of pET28a-Cas9-His plasmid.

	CCACGTTTCTGCGAAAACGCGGGAAAAAGTGGAAGCGGCGATGGCGGAGCTGAATTACATTCCCAAC<br>CGCGTGGCACAACAACTGGCGGGCAAACAGTCGTTGCTGATTGGCGTTGCCACCTCCAGTCTGGCCC<br>TGCACGCGCCGTCGCAAATTGTCGCGGCGATTAAATCTCGCGCCGATCAACTGGGTGCCAGCGTGGT<br>GGTGTCGATGGTAGAACGAAGCGGCGTCGAAGCCTGTAAAGCGGCGGTGCACAATCTTCTCGCGCAA<br>CGCGTCAGTGGGCTGATCATTAACTATCCGCTGGATGACCAGGATGCCATTGCTGTGGAAGCTGCCTG<br>CACTAATGTTCCGGCGTTATTTCTTGATGTCTCTGACCAGACACCCATCAACAGTATTATTTTCTCCCAT<br>GAAGACGGTACGCGACTGGGCGTGGAGCATCTGGTCGCATTGGGTCACCAGCAAATCGCGCTGTTAG<br>CGGGCCCATTAAGTTCTGTCTCGGCGCGTCTGCGTCTGGCTGGCTGGCATAAATATCTCACTCGCAAT<br>CAAATTCAGCCGATAGCGGAACGGGAAGGCGACTGGAGTGCCATGTCCGGTTTTCAACAAACCATGCA<br>AATGCTGAATGAGGGCATCGTTCCCACTGCGATGCTGGTTGCCAACGATCAGATGGCGCTGGGCGCA<br>ATGCGCGCCATTACCGAGTCCGGGCTGCGCGTTGGTGCGGATATCTCGGTAGTGGGATACGACGATA<br>CCGAAGACAGCTCATGTTATATCCCGCCGTTAACCACCATCAAACAGGATTTTCGCCTGCTGGGGCAA<br>ACCAGCGTGGACCGCTTGCTGCAACTCTCTCAGGGCCAGGCGGTGAAGGGCAATCAGCTGTTGCCCG<br>TCTCACTGGTGAAAAGAAAAACCACCCTGGCGCCCAATACGCAAACCGCCTCTCCCCGCGCGTTGGC<br>CGATTCATTAATGCAGCTGGCACGACAGGTTTCCCGACTGGAAAGCGGGCAGTGAGCGCAACGCAAT<br>TAATGTAAGTTAGCTCACTCATTAGGCACCGGGATCTCGACCGATGCCCTTGAGAGCCTTCAACCCAG<br>TCAGCTCCTTCCGGTGGGCGCGGGGCATGACTATCGTCGCCGCACTTATGACTGTCTTCTTTATCATG<br>CAACTCGTAGGACAGGTGCCGGCAGCGCTCTGGGTCATTTTCGGCGAGGACCGCTTTCGCTGGAGCG<br>CGACGATGATCGGCCTGTCGCTTGCGGTATTCGGAATCTTGCACGCCCTCGCTCAAGCCTTCGTCACT<br>GGTCCCGCCACCAAACGTTTCGGCGAGAAGCAGGCCATTATCGCCGGCATGGCGGCCCCACGGGTG<br>CGCATGATCGTGCTCCTGTCGTTGAGGACCCGGCTAGGCTGGCGGGGTTGCCTTACTGGTTAGCAGA<br>ATGAATCACCGATACGCGAGCGAACGTGAAGCGACTGCTGCTGCAAAACGTCTGCGACCTGAGCAAC<br>AACATGAATGGTCTTCGGTTTCCGTGTTTCGTAAAGTCTGGAAACGCGGAAGTCAGCGCCCTGCACCA<br>TTATGTTCCGGATCTGCATCGCAGGATGCTGCTGGCTACCCTGTGGAACACCTACATCTGTATTAACGA<br>AGCGCTGGCATTGACCCTGAGTGATTTTTCTCTGGTCCCGCCGCATCCATACCGCCAGTTGTTTACCC<br>TCACAACGTTCCAGTAACCGGGCATGTTCATCATCAGTAACCCGTATCGTGAGCATCCTCTCTCGTTTC<br>ATCGGTATCATTACCCCCATGAACAGAAATCCCCCTTACACGGAGGCATCAGTGACCAAACAGGAAAA<br>AACCGCCCTTAACATGGCCCGCTTTATCAGAAGCCAGACATTAACGCTTCTGGAGAAACTCAACGAGC<br>TGGACGCGGATGAACAGGCAGACATCTGTGAATCGCTTCACGACCACGCTGATGAGCTTTACCGCAG<br>CTGCCTCGCGCGTTTCGGTGATGACGGTGAAAACCTCTGACACATGCAGCTCCCGGAGACGGTCACA<br>GCTTGTCTGTAAGCGGATGCCGGGAGCAGACAAGCCCGTCAGGGCGCGTCAGCGGGTGTTGGCGGG<br>TGTCGGGGCGCAGCCATGACCCAGTCACGTAGCGATAGCGGAGTGTATACTGGCTTAACTATGCGGC<br>ATCAGAGCAGATTGTACTGAGAGTGCACCATTGCGGTGTGAAATACCGCACAGATGCGTAAGGAGAAA<br>ATACCGCATCAGGCGCTCTTCCGCTTCCTCGCTCACTGACTCGCTGCGCTCGGTCGTTCGGCTGCGG<br>CGAGCGGTATCAGCTCACTCAAAGGCGGTAATACGGTTATCCACAGAATCAGGGGATAACGCAGGAAA<br>GAACATGTGAGCAAAAGGCCAGCAAAAGGCCAGGAACCGTAAAAAGGCCGCGTTGCTGGCGTTTTTC<br>CATAGGCTCCGCCCCCCTGACGAGCATCACAAAAATCGACGCTCAAGTCAGAGGTGGCGAAACCCGA<br>CAGGACTATAAAGATACCAGGCGTTTCCCCCTGGAAGCTCCCTCGTGCGCTCTCCTGTTCCGACCCTG<br>CCGCTTACCGGATACCTGTCCGCCTTTCTCCCTTCGGGAAGCGTGGCGCTTTCTCATAGCTCACGCTG<br>TAGGTATCTCAGTTCGGTGTAGGTCGTTCGCTCCAAGCTGGGCTGTGTGCACGAACCCCCCGTTCAGC<br>CCGACCGCTGCGCCTTATCCGGTAACTATCGTCTTGAGTCCAACCCGGTAAGACACGACTTATCGCCA<br>CTGGCAGCAGCCACTGGTAACAGGATTAGCAGAGCGAGGTATGTAGGCGGTGCTACAGAGTTCTTGA<br>AGTGGTGGCCTAACTACGGCTACACTAGAAGGACAGTATTTGGTATCTGCGCTCTGCTGAAGCCAGTT<br>ACCTTCGGAAAAAGAGTTGGTAGCTCTTGATCCGGCAAACAAACCACCGCTGGTAGCGGTGGTTTTTT<br>TGTTTGCAAGCAGCAGATTACGCGCAGAAAAAAAGGATCTCAAGAAGATCCTTTGATCTTTTCTACGGG<br>GTCTGACGCTCAGTGGAACGAAAACTCACGTTAAGGGATTTTGGTCATGAACAATAAAACTGTCTGCTT<br>ACATAAACAGTAATACAAGGGGTGTTATGAGCCATATTCAACGGGAAACGTCTTGCTCTAGGCCGCGA<br>TTAAATTCCAACATGGATGCTGATTTATATGGGTATAAATGGGCTCGCGATAATGTCGGGCAATCAGGT<br>GCGACAATCTATCGATTGTATGGGAAGCCCGATGCGCCAGAGTTGTTTCTGAAACATGGCAAAGGTAG<br>CGTTGCCAATGATGTTACAGATGAGATGGTCAGACTAAACTGGCTGACGGAATTTATGCCTCTTCCGAC<br>CATCAAGCATTTTATCCGTACTCCTGATGATGCATGGTTACTCACCACTGCGATCCCCGGGAAAACAGC<br>ATTCCAGGTATTAGAAGAATATCCTGATTCAGGTGAAAATATTGTTGATGCGCTGGCAGTGTTCCTGCG<br>CCGGTTGCATTCGATTCCTGTTTGTAATTGTCCTTTTAACAGCGATCGCGTATTTCGTCTCGCTCAGGC<br>GCAATCACGAATGAATAACGGTTTGGTTGATGCGAGTGATTTTGATGACGAGCGTAATGGCTGGCCTG<br>TTGAACAAGTCTGGAAAGAAATGCATAAACTTTTGCCATTCTCACCGGATTCAGTCGTCACTCATGGTG<br>ATTTCTCACTTGATAACCTTATTTTTGACGAGGGGAAATTAATAGGTTGTATTGATGTTGGACGAGTCGG<br>AATCGCAGACCGATACCAGGATCTTGCCATCCTATGGAACTGCCTCGGTGAGTTTTCTCCTTCATTACA<br>GAAACGGCTTTTTCAAAAATATGGTATTGATAATCCTGATATGAATAAATTGCAGTTTCATTTGATGCTC<br>GATGAGTTTTTCTAAGAATTAATTCATGAGCGGATACATATTTGAATGTATTTAGAAAAATAAACAAATAG<br>GGGTTCCGCGCACATTTCCCCGAAAAGTGCCACCTAAATTGTAAGCGTTAATATTTTGTTAAAATTCGC<br>GTTAAATTTTTGTTAAATCAGCTCATTTTTTAACCAATAGGCCGAAATCGGCAAAATCCCTTATAAATCAA<br>AAGAATAGACCGAGATAGGGTTGAGTGTTGTTCCAGTTTGGAACAAGAGTCCACTATTAAAGAACGTG<br>GACTCCAACGTCAAAGGGCGAAAAACCGTCTATCAGGGCGATGGCCCACTACGTGAACCATCACCCTA<br>ATCAAGTTTTTTGGGGTCGAGGTGCCGTAAAGCACTAAATCGGAACCCTAAAGGGAGCCCCCGATTTA<br>GAGCTTGACGGGGAAAGCCGGCGAACGTGGCGAGAAAGGAAGGGAAGAAAGCGAAAGGAGCGGGC<br>GCTAGGGCGCTGGCAAGTGTAGCGGTCACGCTGCGCGTAACCACCACACCCGCCGCGCTTAATGCG<br>CCGCTACAGGGCGCGT

Table S3. Components of culture media for bacterial expression of Cas9$_{aha}$.

1L culture media	Volume/Amounts
M9 minimal medium	200 mL
20% glucose	20 mL
200 mM $MgSO_4$	10 mL
20 mM $CaCl_2$	5 mL
0.5 M $MnCl_2$	0.1 mL
1 mM $FeCl_2$	0.1 mL
DW	765 mL
Arg, His, Ile, Leu, Glu, Gly, Lys, Phe, Cys, Asp, Trp, Pro	50 mg ea
Val	100 mg
Ser, Thr	250 mg ea
Tyr	50 mg
thiamine	5 mg
nicotinamide	1 mg
folic acid	1 mg
choline chloride	1 mg
riboflavin	0.1 mg

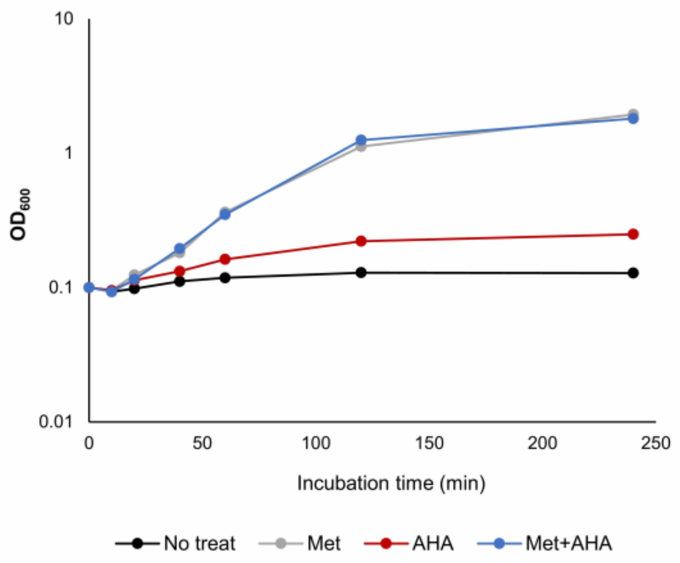

Figure S1. Growth curve of B834(DE3) cultured in presence of Met, AHA, or both Met and AHA (Met+AHA).

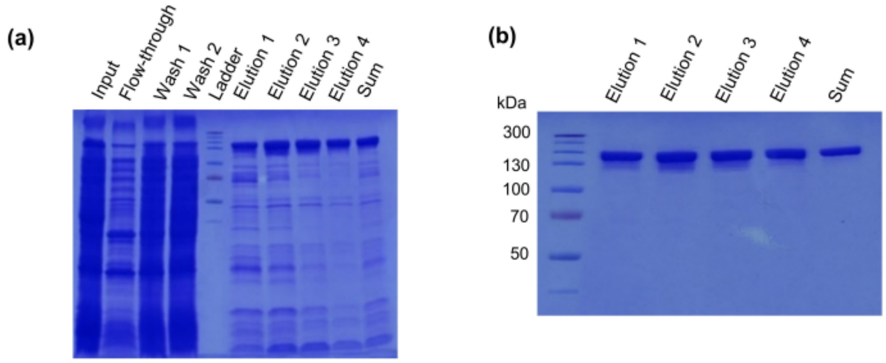

Figure S2. Purification of Cas9$_{aha}$. SDS-PAGE analysis during a) affinity chromatography and b) size exclusion chromatography.

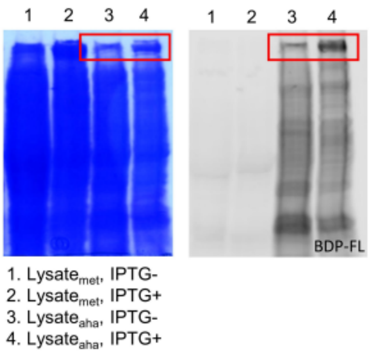

Figure S3. Bioorthogonal reactivity of bacterial lysates after IPTG induction. Lysates from B834(DE3) were reacted with DBCO-BDP-FL and SDS-PAGE was performed for analysis by coomassie blue staining (left) and fluorescence detection (right). Bacterial lysates from growth in Met before IPTG induction (Lane 1); growth in Met after IPTG induction (Lane 2); growth in AHA before IPTG induction (Lane 3); growth in AHA after IPTG induction (Lane 4).

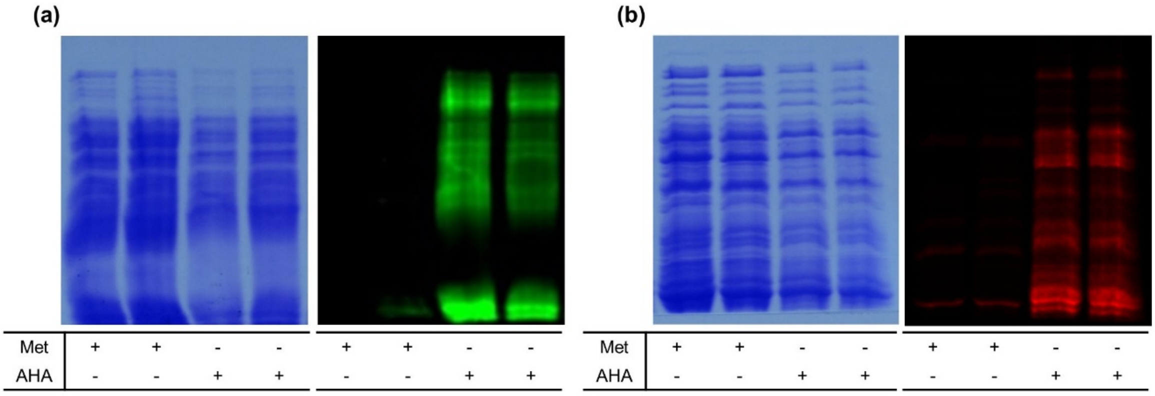

Figure S4. Bioorthogonal reactivity of bacterial lysates from cultured B834(DE3) by strain-promoted azide-alkyne cycloadditions a) and copper-mediated azide-alkyne cycloaddition b). Analysis by SDS-PAGE and coomassie blue staining (left) and fluorescence detection (right). Bacterial lysates from growth in the presence of Met (Lanes 1,2), and AHA (Lanes 3,4).

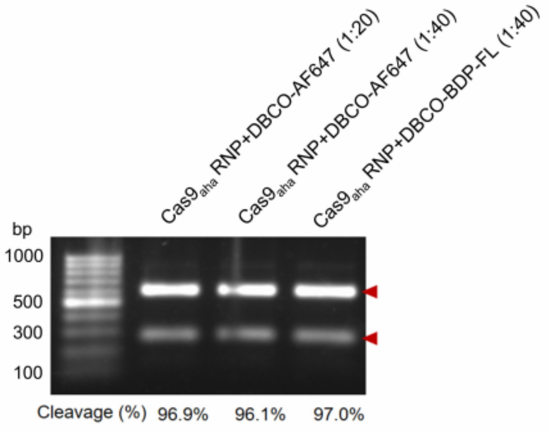

Figure S5. *In vitro* cleavage assay of Cas9$_{aha}$ RNPs reacted with DBCO-functionalized dye. Cas9$_{aha}$ was reacted with DBCO-dye and complexed with sgRNA-1.

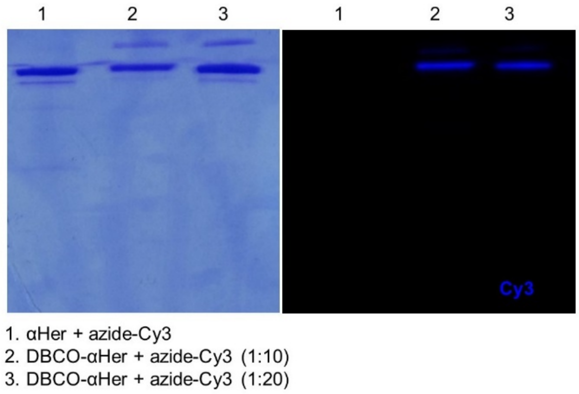

Figure S6. Bioorthogonal reactivity of DBCO-αHer. SDS-PAGE of αHer (lane 1) and DBCO-αHer reacted with azide-Cy3 (molar ratios of 1:10 for lane 2; 1:20 for lane 3), and analysis by coomassie blue staining (left) and fluorescence detection (right).

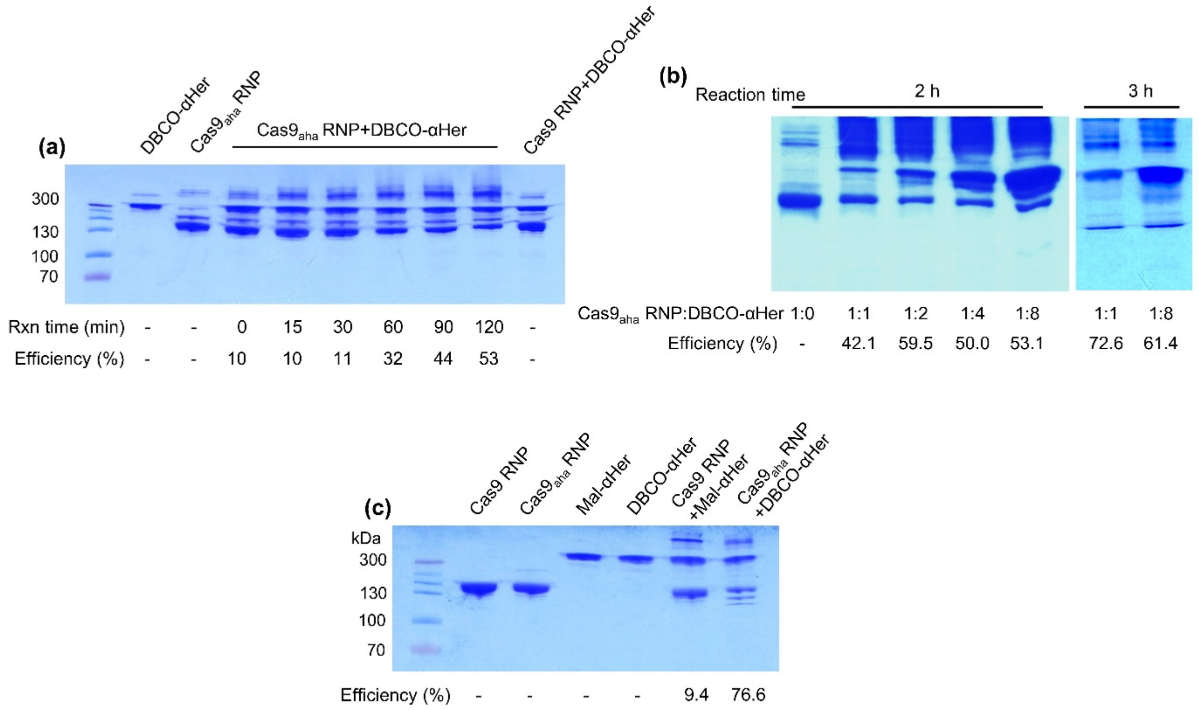

Figure S7. Characterization of $Cas9_{aha}$ RNPs reacted with DBCO-αHer by SDS-PAGE. a) Various incubation times used for reaction, b) Conjugation efficiency depending on the molar ratio of $Cas9_{aha}$ RNP:DBCO-αHer, and c) comparison with native Cas9 RNPs reacted with Mal-αHer (Cas9 RNP+Mal-αHer). Conjugation efficiencies were calculated by image analysis of band intensities and labeled below.

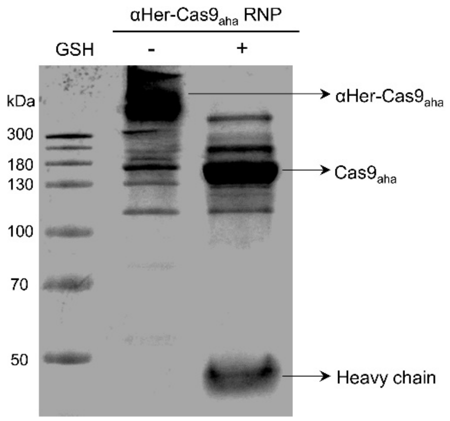

Figure S8. Analysis of αHer-Cas9$_{aha}$ RNPs incubated in reducing condition (5 mM GSH) by SDS-PAGE and Coomassie blue staining.

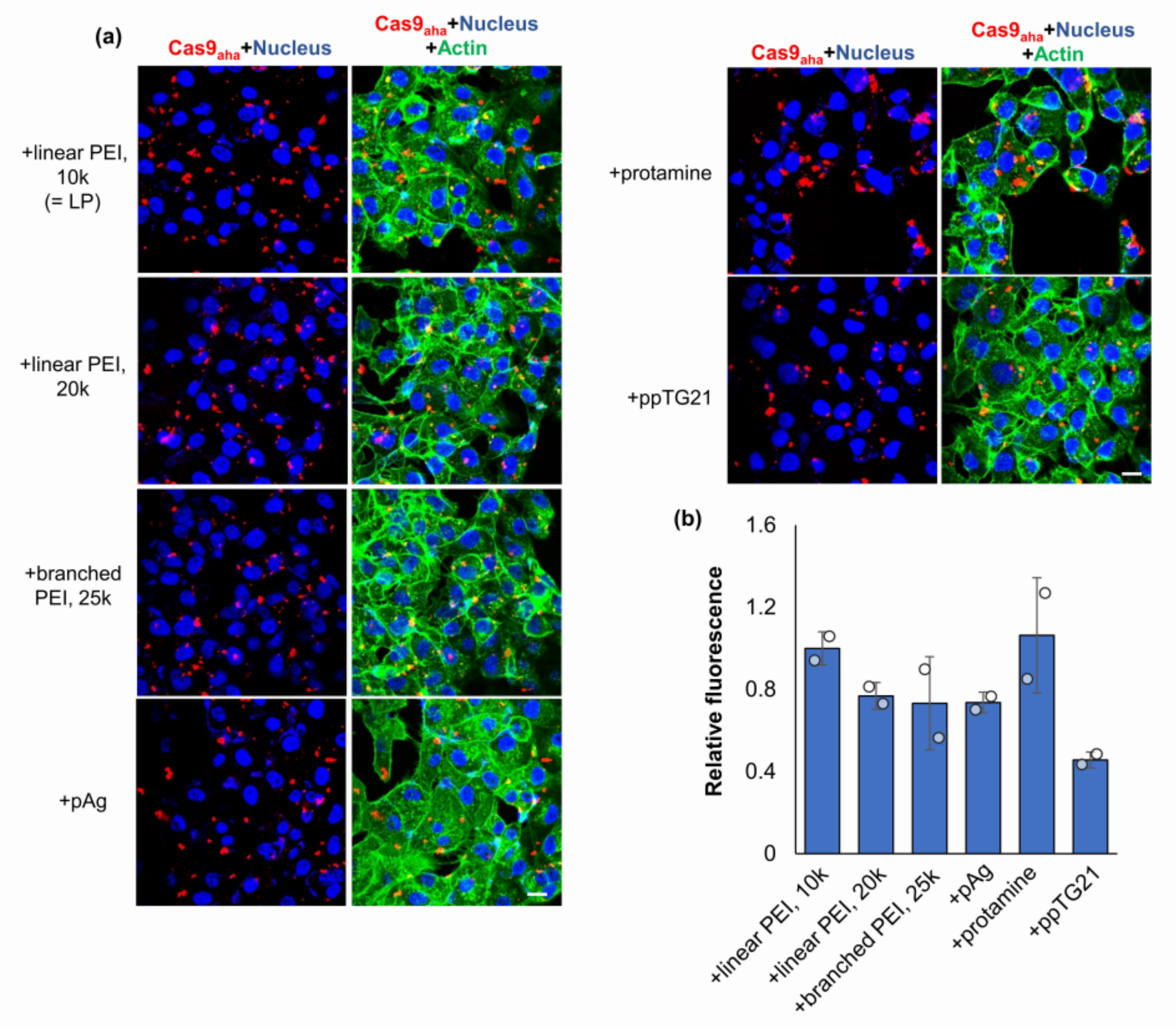

Figure S9. Internalization by treating Cas9$_{aha}$ RNPs labeled with AF647 complexed with polyethylenimine (PEI, linear and branched type), polyarginine (pAg), protamine, or ppTG21, followed by conjugation with αHer to SKOV3 cells, and observed by confocal microscopy. (40X magnification, scale bar: 20 μm) b) Quantification of fluorescence signals (bars represent mean ± S.D).

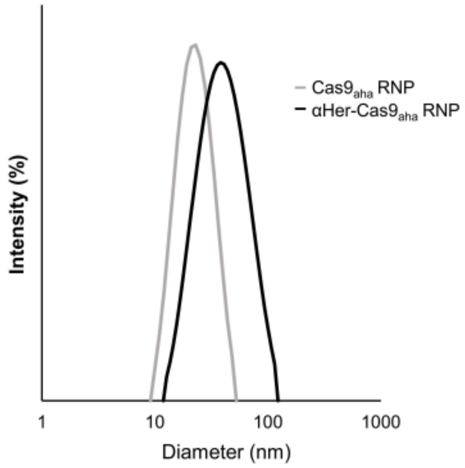

Figure S10. Hydrodynamic diameters of Cas9$_{aha}$ RNPs and αHer-Cas9$_{aha}$ RNPs.

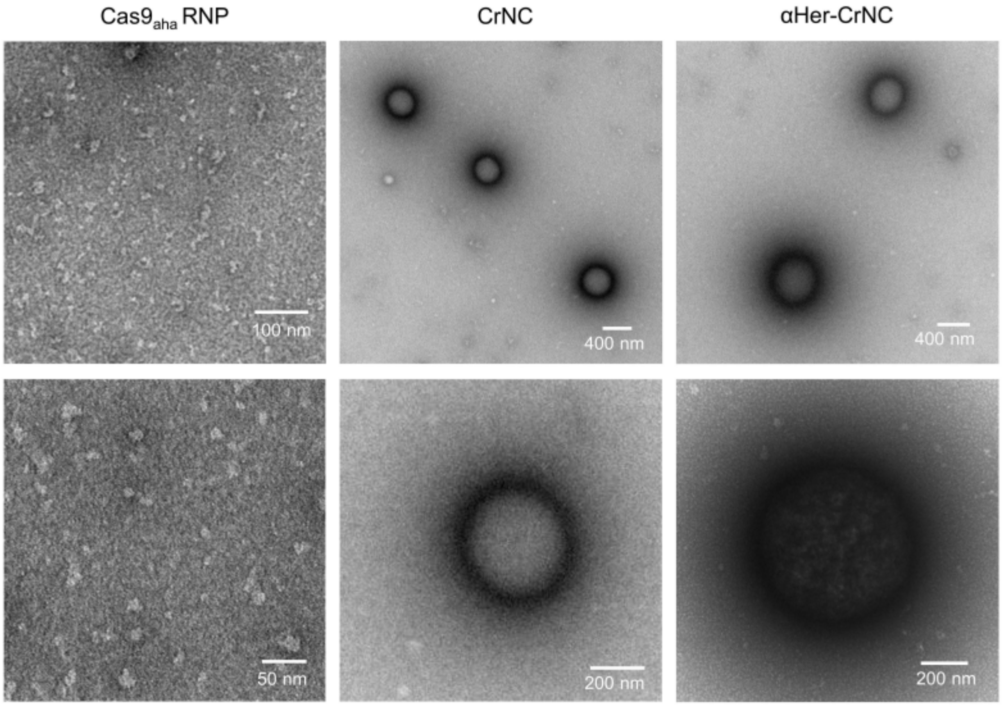

Figure S11. TEM analysis of $Cas9_{aha}$ RNPs (left), CrNC (middle), and αHer-CrNC (right). Samples were stained by uranyl acetate.

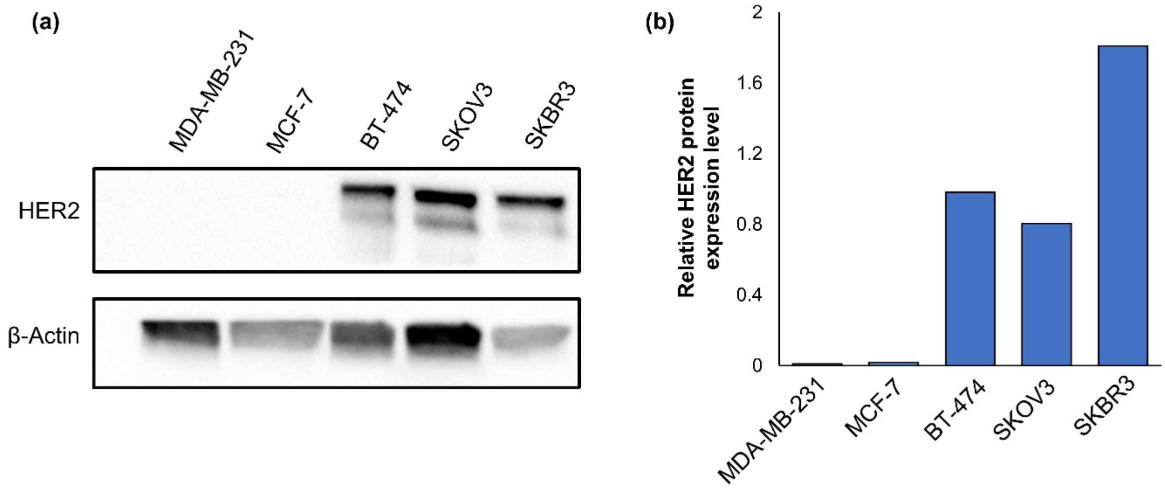

Figure S12. Expression level of HER2 from various cancer cells. MDA-MB-231 and MCF-7 are HER2-negative cells, and BT-474, SKOV3 and SKBR3 are HER2-positive cancer cells. a) Western blot, and b) quantification of a).

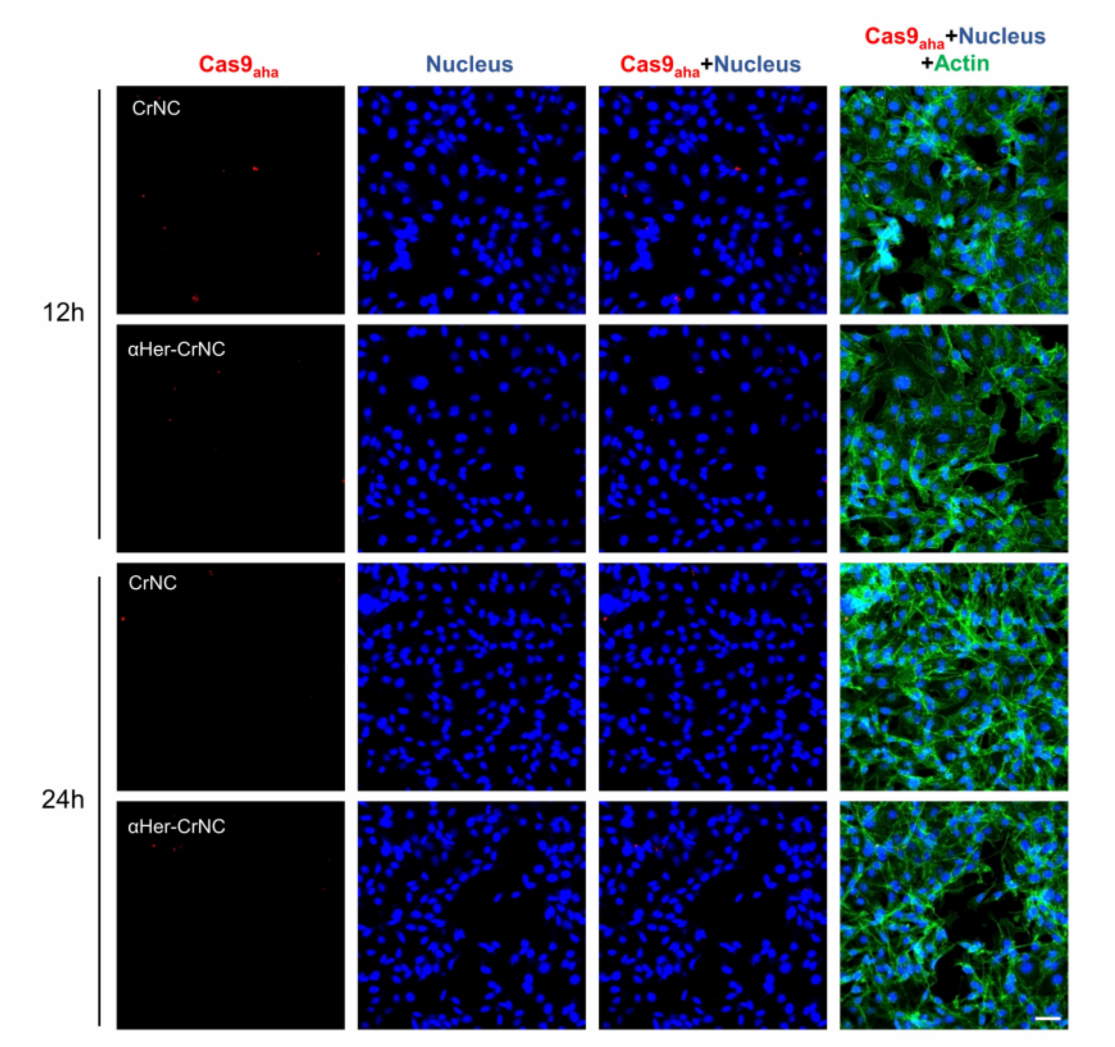

Figure S13. Treatment of αHer-CrNC to MDA-MB-231 cells for the indicated incubation times and confocal microscopy. (red: Cas9$_{aha}$; blue: nucleus; green: actin, 20X magnification, scale bar: 50 μm)

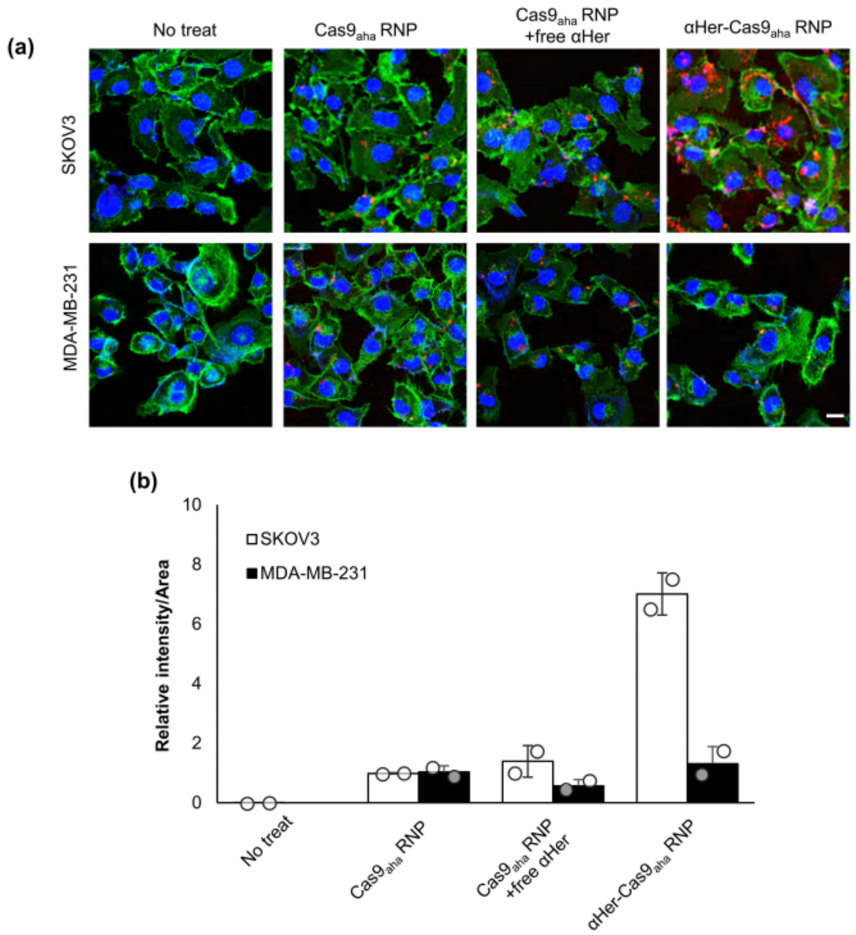

Figure S14. Examination of cellular uptake by treating αHer-Cas9$_{aha}$ RNPs. a) Confocal images of the treated SKOV3 and MDA-MB-231 cells (red: AF647-labeled Cas9$_{aha}$; blue: DAPI; green: actin, 40X magnification, scale bar: 20 μm), and b) quantification of fluorescence signals (bars represent mean ± S.D).

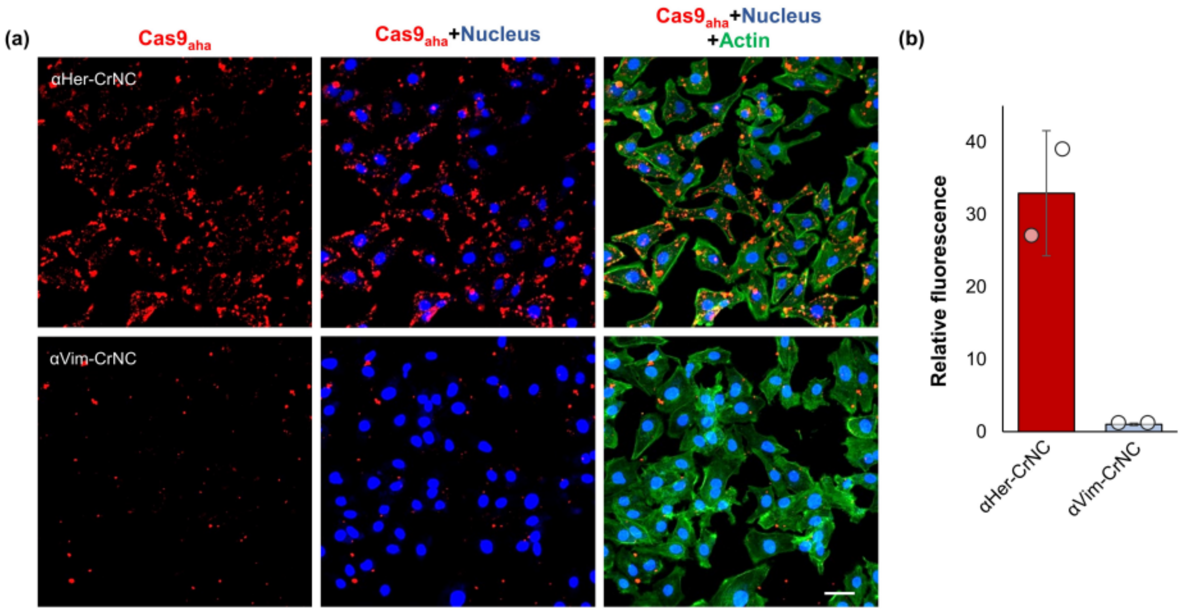

Figure S15. Internalization of αHer-CrNC compared with complexes conjugated with non-target antibody (αVim-CrNC) into SKOV3 cells. a) Images from confocal microscopy (20X magnification, scale bar: 50 μm), and b) quantification of fluorescence from images in a).

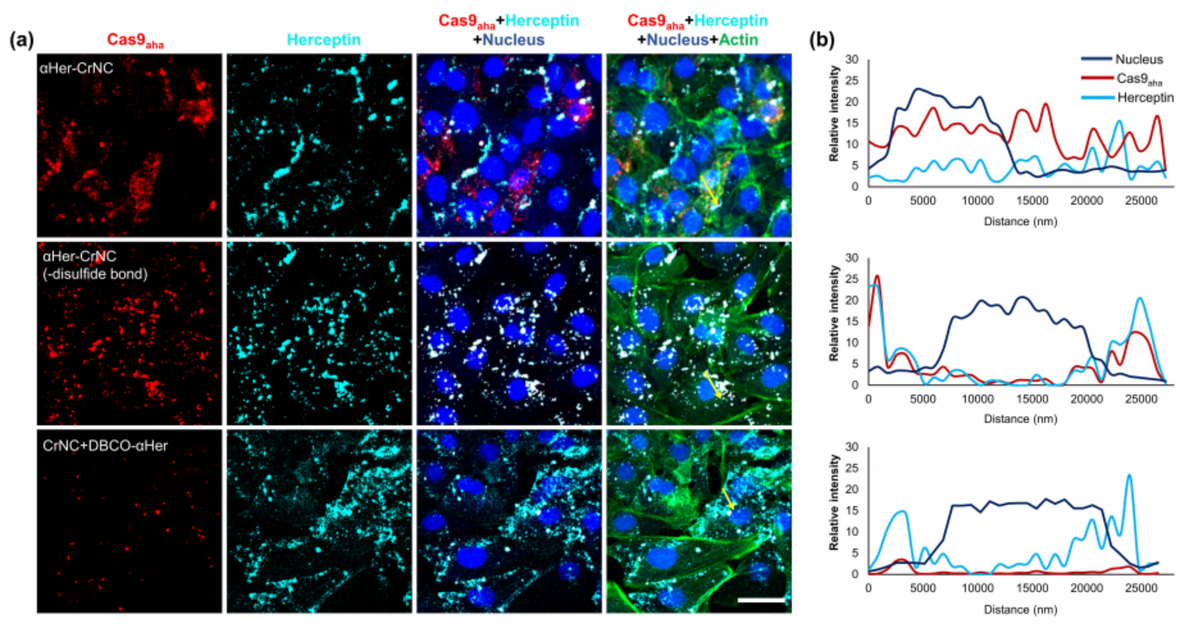

Figure S16. Examination of co-localization of $Cas9_{aha}$ and Herceptin in SKOV3 cells treated with αHer-CrNC. a) Confocal images of the treated cells (red: $Cas9_{aha}$; cyan: Herceptin; blue: DAPI; green: actin, 40X magnification, scale bar: 50 μm), and b) relative fluorescence intensity scan along the arrow line indicated in a).

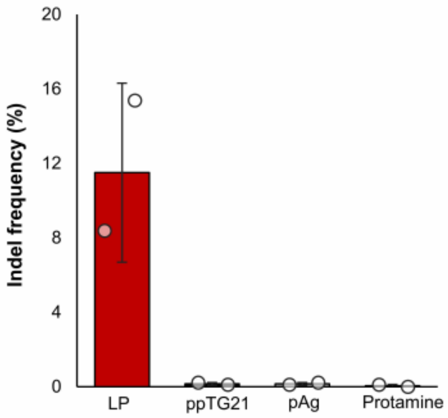

Figure S17. Indel frequency by treating $Cas9_{aha}$ RNPs complexed with different peptides and polymers, followed by conjugation with αHer to SKOV3 cells (LP: linear polyethylenimine, Mw 10 kDa; ppTG21: endosomolytic peptide; pAg: polyarginine) (bars represent mean ± S.D).

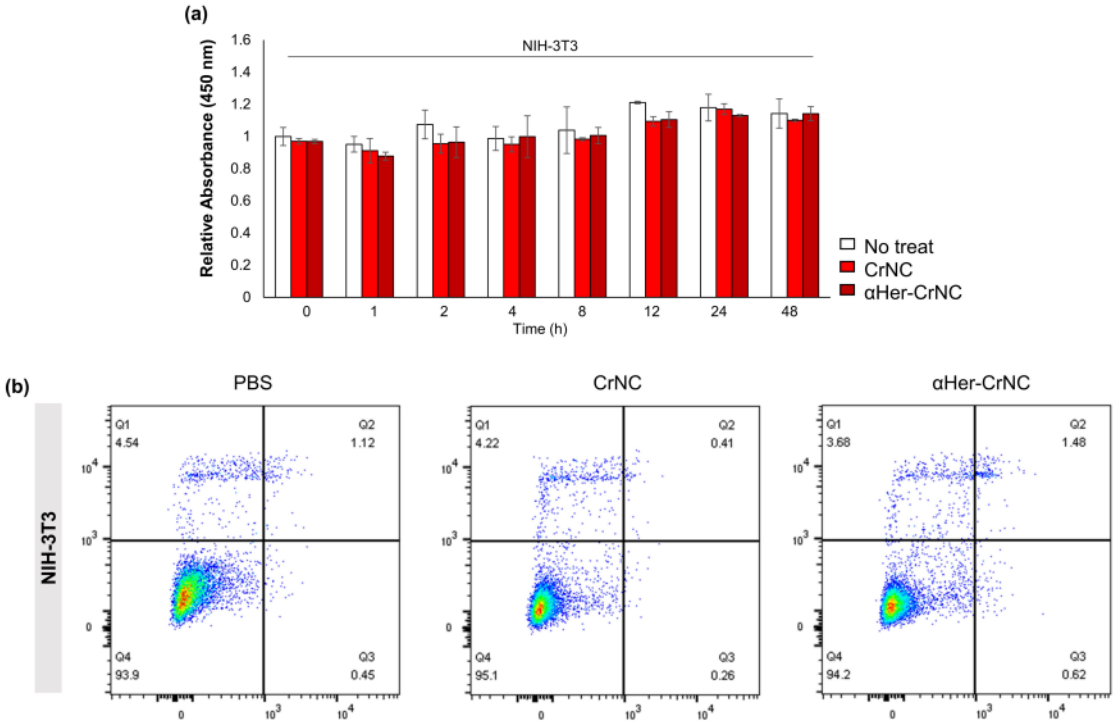

Figure S18. a) Cytotoxicity and b) apoptosis assay of treating CrNC and αHer-CrNC to NIH-3T3 cells (bars represent mean ± S.D).

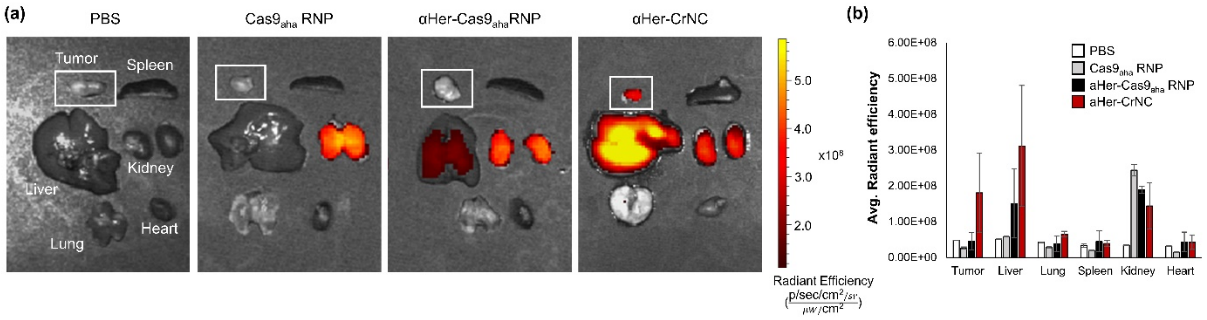

Figure S19. a) Biodistribution of αHer-CrNC and controls after intravenous injection, by detecting AF750 the fluorescence of Cas9$_{aha}$ (24 h post-injection), and b) quantification of average radiant efficiency from a).

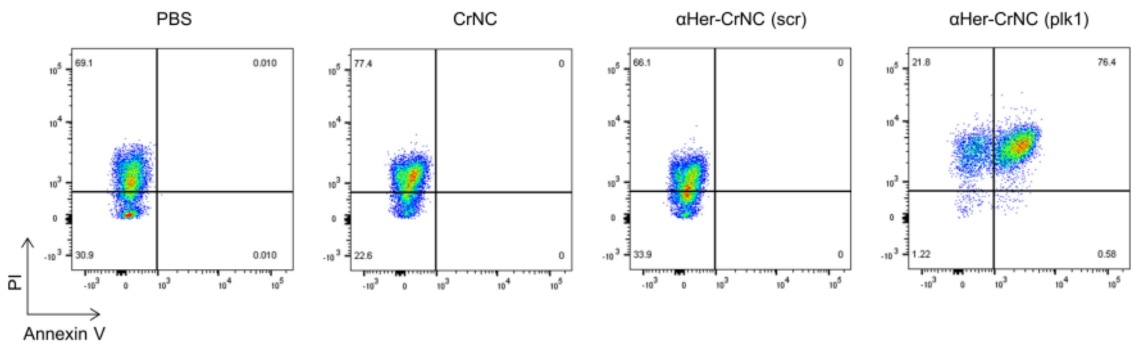

Figure S20. Apoptosis levels of tumors after *in vivo* delivery of complexes. αHer-CrNC (plk1) and the control complexes were treated to SKOV3 tumors in mice, harvested on day 19, and analyzed by the Annexin V/PI apoptosis assay.
